# Deep learning model for diagnosing early gastric cancer using preoperative computed tomography images

**DOI:** 10.3389/fonc.2022.1065934

**Published:** 2022-11-30

**Authors:** Qingwen Zeng, Zongfeng Feng, Yanyan Zhu, Yang Zhang, Xufeng Shu, Ahao Wu, Lianghua Luo, Yi Cao, Jianbo Xiong, Hong Li, Fuqing Zhou, Zhigang Jie, Yi Tu, Zhengrong Li

**Affiliations:** ^1^ Department of Gastrointestinal Surgery, The First Affiliated Hospital, Nanchang University, Nanchang, Jiangxi, China; ^2^ Institute of Digestive Surgery, The First Affiliated Hospital of Nanchang University, Nanchang, Jiangxi, China; ^3^ Medical Innovation Center, The First Affiliated Hospital of Nanchang University, Nanchang, China; ^4^ Department of Radiology, The First Affiliated Hospital, Nanchang University, Nanchang, Jiangxi, China; ^5^ Department of Radiology, The Second Affiliated Hospital of Soochow University, Suzhou, Jiangsu, China; ^6^ Department of Pathology, The First Affiliated Hospital of Nanchang University, Nanchang, Jiangxi, China

**Keywords:** early gastric cancer, deep learning, computed tomography, convolutional neural, diagnosis

## Abstract

**Background:**

Early gastric cancer (EGC) is defined as a lesion restricted to the mucosa or submucosa, independent of size or evidence of regional lymph node metastases. Although computed tomography (CT) is the main technique for determining the stage of gastric cancer (GC), the accuracy of CT for determining tumor invasion of EGC was still unsatisfactory by radiologists. In this research, we attempted to construct an AI model to discriminate EGC in portal venous phase CT images.

**Methods:**

We retrospectively collected 658 GC patients from the first affiliated hospital of Nanchang university, and divided them into training and internal validation cohorts with a ratio of 8:2. As the external validation cohort, 93 GC patients were recruited from the second affiliated hospital of Soochow university. We developed several prediction models based on various convolutional neural networks, and compared their predictive performance.

**Results:**

The deep learning model based on the ResNet101 neural network represented sufficient discrimination of EGC. In two validation cohorts, the areas under the curves (AUCs) for the receiver operating characteristic (ROC) curves were 0.993 (95% CI: 0.984-1.000) and 0.968 (95% CI: 0.935-1.000), respectively, and the accuracy was 0.946 and 0.914. Additionally, the deep learning model can also differentiate between mucosa and submucosa tumors of EGC.

**Conclusions:**

These results suggested that deep learning classifiers have the potential to be used as a screening tool for EGC, which is crucial in the individualized treatment of EGC patients.

## Background

Gastric cancer (GC) is one of the most primary cancers and ranked as fifth and fourth in the global incidence rate and mortality rate in 2020 (1). The prognosis of GC was closed associated with the depth of invasion, because the patients with advanced GC had a 5-year survival rate of less than 30%, however, the patients with early gastric cancer (EGC) were more than 90% (2, 3). EGC is defined as a lesion restricted to the mucosa or submucosa, independent of size or evidence of regional lymph node metastases. Accurate preoperative diagnosis at an early stage of GC provides the greatest prognosis and is critical for planning effective therapy, such as endoscopic submucosal dissection (ESD), endoscopic mucosal resection (EMR) and laparoscopic surgery (4, 5). Notably, the early identification and precise preoperative staging of EGC are particularly crucial.

For the preoperative diagnosis of EGC, computed tomography (CT) and endoscopic ultrasonography (EUS) are the most commonly used methods. The main tumor can be seen on a CT scan, which can also measure the extent of tumor invasion and find nodal involvement and distant metastases (6). According to reports, CT’s overall diagnostic accuracy for T-staging ranges from 73.8% to 88.9%. Radiologist evaluation of its T1 stage diagnostic accuracy, however, was average from 63% to 82.7% (7, 8). In certain postoperative cases of EGC (9, 10), it was discovered, based on several published research and our experience, that some EGC was over-staged as advanced GC in clinical practice. As a result, the majority of EGC patients underwent an excessive amount of D2 lymphadenectomy therapy (11). There have been many studies on the diagnostic power of EUS, because it allows for a clear view of the various layers of the stomach wall, making it one of the most helpful techniques for T staging (12, 13). According to prior research, for T1 staging, individual EUS accuracy varied from 14 to 100%, and the pooled accuracy was 77% (95 CI: 70-84%) (14, 15). However, due to the limits of medical technology and equipment, EUS can only be carried out in reputable medical facilities. Additionally, various tumor sites and stenosis of natural orifices also reduced the effectiveness of EUS for staging, particularly at the gastroesophageal junction (16). This pushes us to come up with a fresh method of enhancing EGC staging diagnosis.

Nowadays, Deep learning has been extensively discussed in the context of medical image analysis, including disease diagnosis, prognosis, and therapy. The deep learning-based models were trained utilizing enormous volumes of data from individuals with known messages, then the accomplished convolutional neural network (CNN) model may utilize data from other persons to predict their likelihood for that occurrence (17). Ole-Johan et al. created a biomarker of patient outcome after primary colorectal cancer resection by directly analyzing scanned conventional haematoxylin and eosin-stained sections using deep learning, which may help doctors make better decisions regarding adjuvant therapy options (18). An international multicenter study, that sought to develop a deep learning radiomic nomogram based on the images from multiphase CT, was successful in distinguishing the number of lymph node metastasis in local GC with excellent accuracy (19). For peritoneal recurrence and prognosis in GC, a multitask prediction models that incorporated preoperative CT images with CNNs demonstrated good accuracy. Adjuvant treatment was linked to increased disease-free survival (DFS) in stage II-III illness for patients with a forecasted high probability of peritoneal recurrence and low survival (20). However, there haven’t been any reports of using CT images along with deep learning to diagnose EGC staging. With the use of portal venous phase CT images, the goal of this work is to build a deep learning model for accurately distinguishing EGC.

## Materials and methods

### Patients

The cohort 1 in this study were 658 GC patients who had surgery in the first affiliated hospital of Nanchang University between June 2018 and December 2021. The training and internal validation cohorts were split up at random from the GC patients of cohort 1 with the ratio of 8:2. As the external validation cohort, 93 GC patients were recruited from the second affiliated hospital of Soochow university from January to December 2021. The following patients were excluded from the study (1): patients with incomplete clinical information after diagnosis (2), patients without preoperative CT images (3), patients whose quality of preoperative CT images was insufficient for further analysis (4), patients who had preoperative CT examinations more than 14 days before surgery (5), patients who had received neoadjuvant chemotherapy. **Supplementary Figure 1** showed the flowchart of inclusion and exclusion criteria for EGC patients for the cohort 1. We employed PASS software to estimate the sample size, and the sample size of the advanced GC was 1.5 times of the EGC patients as control group (**Supplementary Figure 2**). According to the purpose of our study, all GC patients were divided into two categories, including EGC and advanced GC, to develop a prediction model for identifying EGC. Advanced GC was defined when the invasion depth exceeded the submucosal level. There were 210 EGC patients and 316 advanced GC patients in the training cohort, 53 EGC patients and 79 advanced GC patients in the internal validation cohort, and 23 EGC patients and 70 advanced GC patients in the external validation cohort. The medical ethics committee of the first affiliated hospital of Nanchang University approved this retrospective study protocol (IRB number: 2022-CDYFYYLK-09-041).

### Image acquisition

For contrast-enhanced CT scanning, the following scanners were used: 128-channel CT (IQon Spectral CT), 256-channel CT (Philips Brilliance iCT 256), 256-channel CT (Siemens Healthcare) and 128-channel CT (Siemens Healthcare). The scanning specifications were as follows: a tube voltage range of 80 to 120 kVp, a tube current range of 120 to 300 mAs, a pitch range of 0.6 to 1.25 mm, an image matrix of 512×512, and a reconstruction slice thickness range of 1 or 2 mm. Before having an abdominal contrast-enhanced CT, each patient had an intramuscular injection of 20 mg of Racanisodamine Hydrochloride and drank 1,000-2,000 mL of water. Following intravenous injection of contrast media (1.5mL/kg, at a rate of 3.0-3.5mL/s), the arterial phase and portal venous phase were recorded in 25-30 seconds and 65-70 seconds, respectively.

### Manual label of tumor and images preprocessing

**Figure 1:**
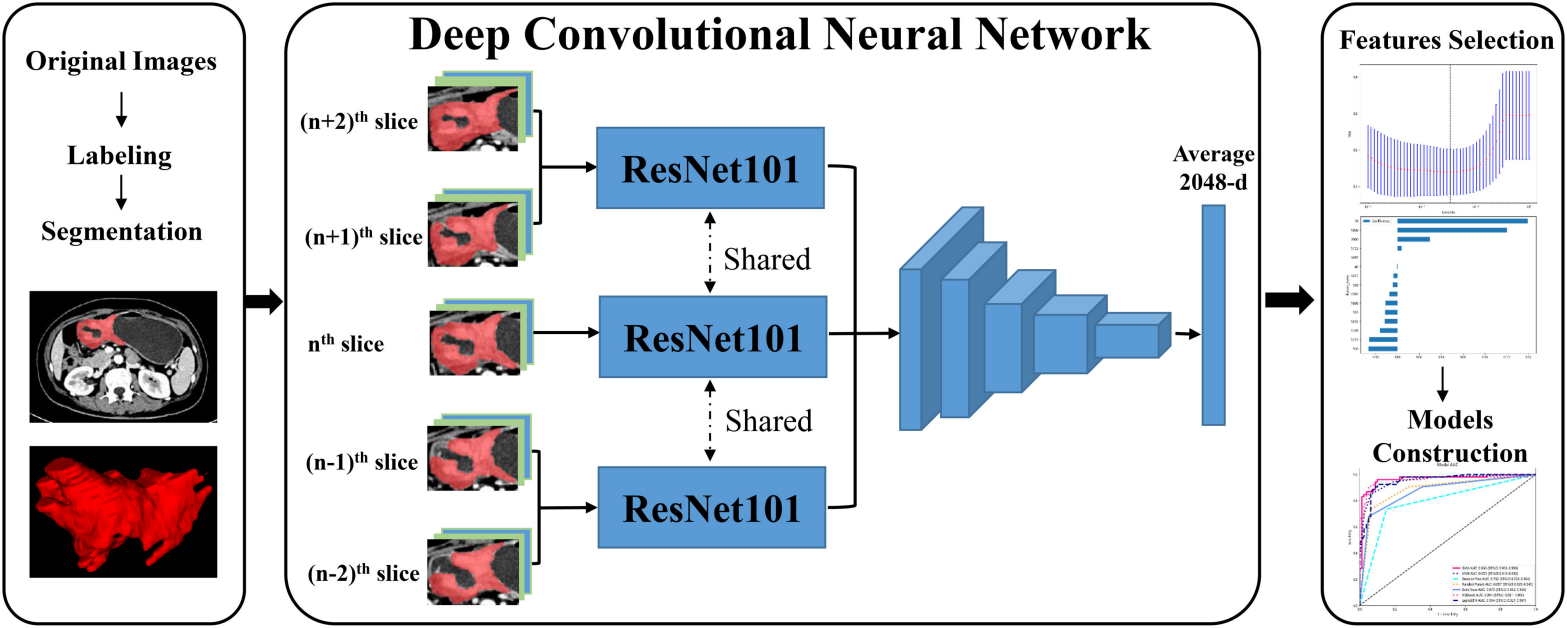
The workflow of entire research design.

During the portal venous phase, the tumor lesion was significantly increased and more easily separated from peripheral normal tissue, and many earlier studies employed this phase to segment tumor lesions (21, 22). The regions of interest (ROIs) of CT images were manually labeled by two radiologists (Z.Y., a junior radiologist and Z.F., a senior radiologist) using ITK-SNAP (version 3.6.0, USA). After the junior radiologist had completed sketching the tumor lesion, the senior radiologist checked the ROI for quality and made a few tweaks. In the three-dimensional (3D) medical imaging, we carefully delineated the adjacent upper and lower slices of the solid tumor, making sure not to include the normal stomach wall or any nearby air or fluid. The radiologist determined the input volume’s n^th^ slice to be the one with the largest tumor lesion. Then, for further analysis, we isolated the (n - 2)^th^, (n - 1)^th^, n^th^, (n + 1)^th^, and (n + 2)^th^ slices. These slices were saved as ‘png’ format. All GC pictures had standardized image contrast based on the abdominal window (window level: 200 HU, window width: 55 HU). The ROI was resized to 224×224 pixels, after being clipped out from these had extracted slices. **Figure 1** displayed the flow chart for the entire research design.

### Deep learning and deep transfer learning features extraction

**Figure 2:**
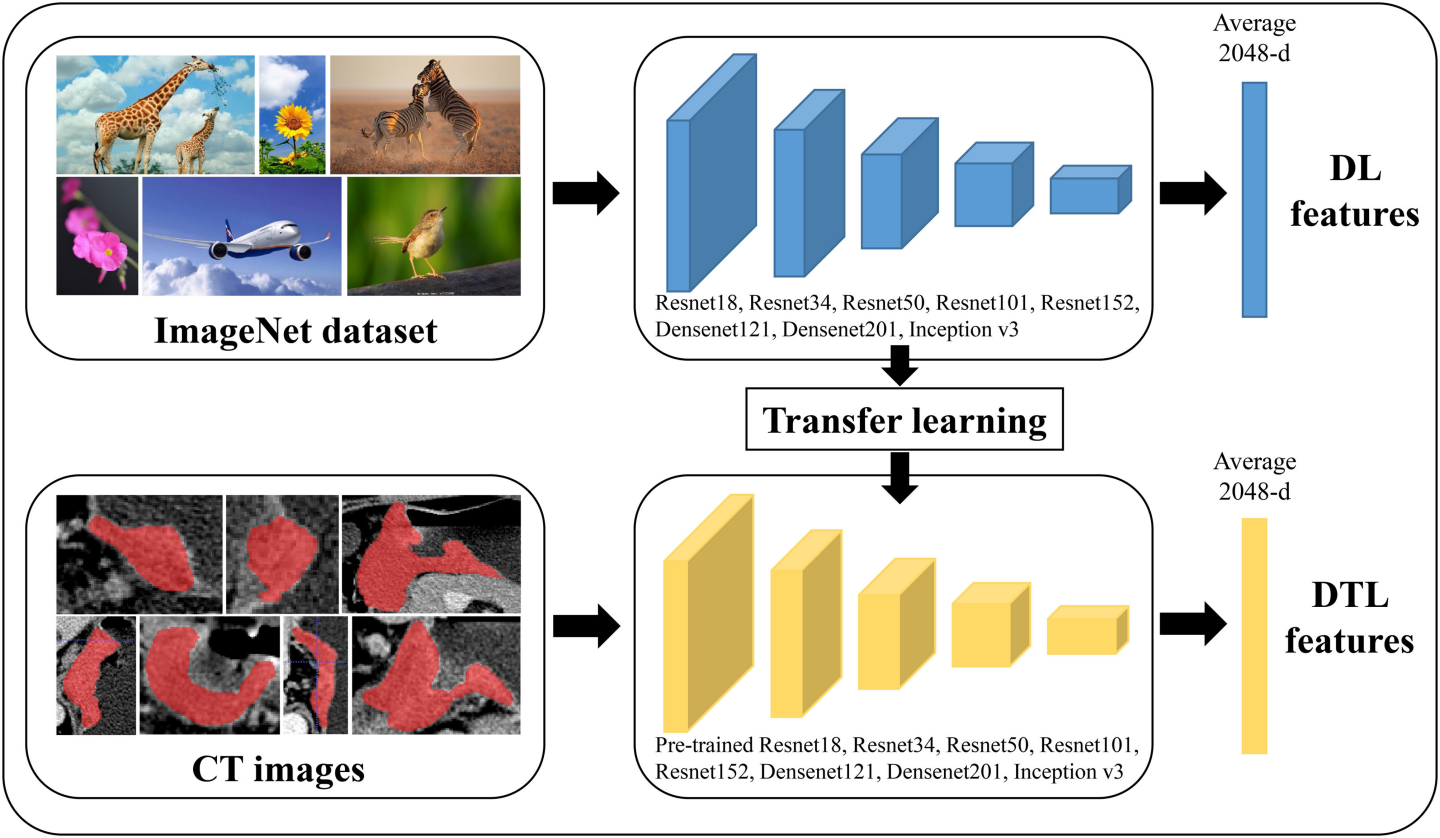
The workflow of deep learning and deep transfer learning (Pre-trained CNNs). DL, deep learning; DTL, deep transfer learning.

In this study, we extracted DL and deep DTL features to constructed a prediction model, respectively. DL features were extracted based on various CNN models from every standardized ROI image, including ResNet18, ResNet34, ResNet50, ResNet101, ResNet152, Densenet121, Densenet201 and inception v3, which were pre-trained based on images of reality. For DTL features, the parameters of these CNN models were pre-trained by all of the ROI images from cohort 1. Then, the pre-trained CNN models were used to extract DTL features for each ROI image. The following process was utilized for all feature acquisition: ROI pictures were input into each CNN model or pre-trained CNN model, the average probability from all images was used to produce DL or DTL features, and the output from the penultimate FC layer was used as DL or DTL features (**Figure 2**). Since every ROI of the (n - 2)^th^, (n - 1)^th^, n^th^, (n + 1)^th^, and (n + 2)^th^ slices were extracted from one GC patients, the DL or DTL features of five slices were averaged to represent each GC patient. Furthermore, our research was implemented in Python 3.10 and run on a system with an Intel Xeon Silver 4214 CPU and 256 GB memory.

### DL and DTL feature prediction model building

Each features group was employed individually to normalize combined features by z score normalization in the training, internal validation and external validation cohorts to merge features of various magnitudes into one magnitude. Non-zero coefficients served as useful predictors in each feature group using the absolute shrinkage and selection operator (LASSO) regression for feature selection in the training cohort (**Supplementary Figure 3**). To create machine learning classification models for multiple feature groups, we used Python Scikit-learn. The performance of several machine learning classifiers, including the support vector machine (SVM), K-nearest neighbor (KNN), decision trees, random decision forests (RF), extra trees, XGBoost and lightGBM was compared using the DeLong test. The receiver operator characteristic (ROC) analysis was used to evaluate the performances of all established models, and the area under the ROC curve (AUC) was used to determine their discriminative ability. A few quantitative metrics were specificity, sensitivity and accuracy. After constructing the classifier for diagnosing EGC, we will verify its generalization ability in the internal validation and external validation cohorts.

### Statistical analysis

Statistical analyses were performed with IBM SPSS Statistics (Version 20.0, USA) for windows. The Student’s *t*-test and analysis of variance were used to analyze the quantitative data, which were reported as mean ± SD. Chi-square test and Fisher exact test were used to analyze the qualitative data. we used the DeLong test to calculate differences between several models using the MedCalc software (version 20.100). A statistically significant difference was determined to exist when *P*-value was less than 0.05. LASSO regression analysis and z score normalization were carried out using Python (version 3.10, available at https://www.python.org/).

## Results

### Demographics of patients

**Table 1** summarized the detailed demographics of GC patients. There were 526, 132 and 93 GC patients enrolled in the training, internal validation and external validation cohorts. The proportions of EGC were 39.92%, 40.15% and 24.73 in the training, internal validation and external validation cohorts. There were 81 invasion of the mucosa (T1a) patients and 129 invasion of the submucosa (T1b) patients in the training cohort, while the internal validation cohort enrolled 19 invasion of the mucosa patients and 34 invasion of the submucosa patients. After statistical analysis by SPSS software, we found that age, tumor size, tumor location, pathological T stage (T1 vs T2-4), lauren’s classification, grade of differentiation, CEA level, CA12-5 level and CA19-9 level were no significantly statistical differences between the training and internal validation cohorts (*P*-value > 0.05), instead of gender. While, age, gender, tumor location, pathological T stage (T1 vs T2-4), lauren’s classification and CA19-9 level were shown significantly statistical differences between the training and external validation cohorts (*P*-value < 0.05). Only tumor size, grade of differentiation, CEA level and CA12-5 level were not significantly different between the training and validation cohorts. This was mainly due to the limited sample size of the external validation cohort.

**Table 1:** Characteristics of GC patient including for classification modeling.

Characteristics	Training cohort (n=526)	Internal validation cohort (n=132)	*P*-value	External validation cohort (n=93)	*P*-value
**Age, mean ± SD, year**	61.33 ± 11.30	60.27 ± 10.81	0.333	67.26 ± 9.54	**<0.001**
**Tumor size, mean ± Std, mm**	37.94 ± 22.86	41.00 ± 25.08	0.178	40.55 ± 27.09	0.325
**Gender, No.(%)**			**0.047**		**<0.001**
Male	313 (59.51)	91 (68.94)		74 (79.57)	
Female	213 (40.49)	41 (31.06)		19 (80.43)	
**Tumor location, No.(%)**			0.316		**0.004**
Upper-third	93 (17.68)	27 (20.46)		27 (29.03)	
Middle-third	158 (30.04)	31 (23.48)		15 (16.13)	
Lower-third	275 (52.28)	74 (56.06)		51 (54.84)	
**Pathological T stage** ^†^ **, No.(%)**			0.962		**0.005**
T1	210 (39.92)	53 (40.15)		23 (24.73)	
T2-4	316 (60.08)	79 (59.85)		70 (75.27)	
**Lauren’s classification, No.(%)**			0.074		**0.006**
Intestinal type	301 (57.22)	69 (52.27)		37 (39.78)	
Diffuse type	97 (18.44)	36 (27.27)		27 (29.04)	
Mixed type	128 (24.34)	27 (20.46)		29 (31.18)	
**Grade of differentiation, No.(%)**			0.604		0.437
Poor/undifferentiated	206 (39.16)	58 (43.94)		43 (46.24)	
Moderate	280 (53.23)	65 (49.24)		44 (47.31)	
Well	40 (7.61)	9 (6.82)		6 (6.45)	
**CEA level, No.(%)**			0.067		0.518
Negative	488 (92.78)	116 (87.88)		88 (94.62)	
Positive	38 (7.22)	16 (12.12)		5 (5.38)	
**CA12-5 level, No.(%)**			0.853		0.382
Negative	504 (95.82)	126 (95.45)		88 (94.62)	
Positive	22 (4.18)	6 (4.55)		5 (5.38)	
**CA19-9 level, No.(%)**			0.342		**0.004**
Negative	445 (84.60)	116 (87.88)		89 (95.70)	
Positive	81 (15.40)	16 (12.12)		4 (4.30)	

Quantitative variables were in mean ± SD and qualitative variables are in n (%). ^†^According to the eighth edition AJCC Cancer Staging Manual. Bold values meant P-value < 0.05.

### Result of the feature extraction and selection

As shown in **Figure 2** and **Supplementary Table 1**, multiple CNN and pre-trained CNN models were used to extract 512-2,048 DL and DTL features for each patient from each ROI slice. All features were examined using LASSO regression and z sore normalization, and all features with non-zero coefficients were chosen to build classification models with the five-fold cross test. For example, in ResNet101 and pre-trained ResNet101 models, there were 85 DL and 15 DTL features selection using LASSO regression. The detail selection features of several CNN models were displayed in **Supplementary Table 2**.

### The performance of different CNN models

**Figure 3:**
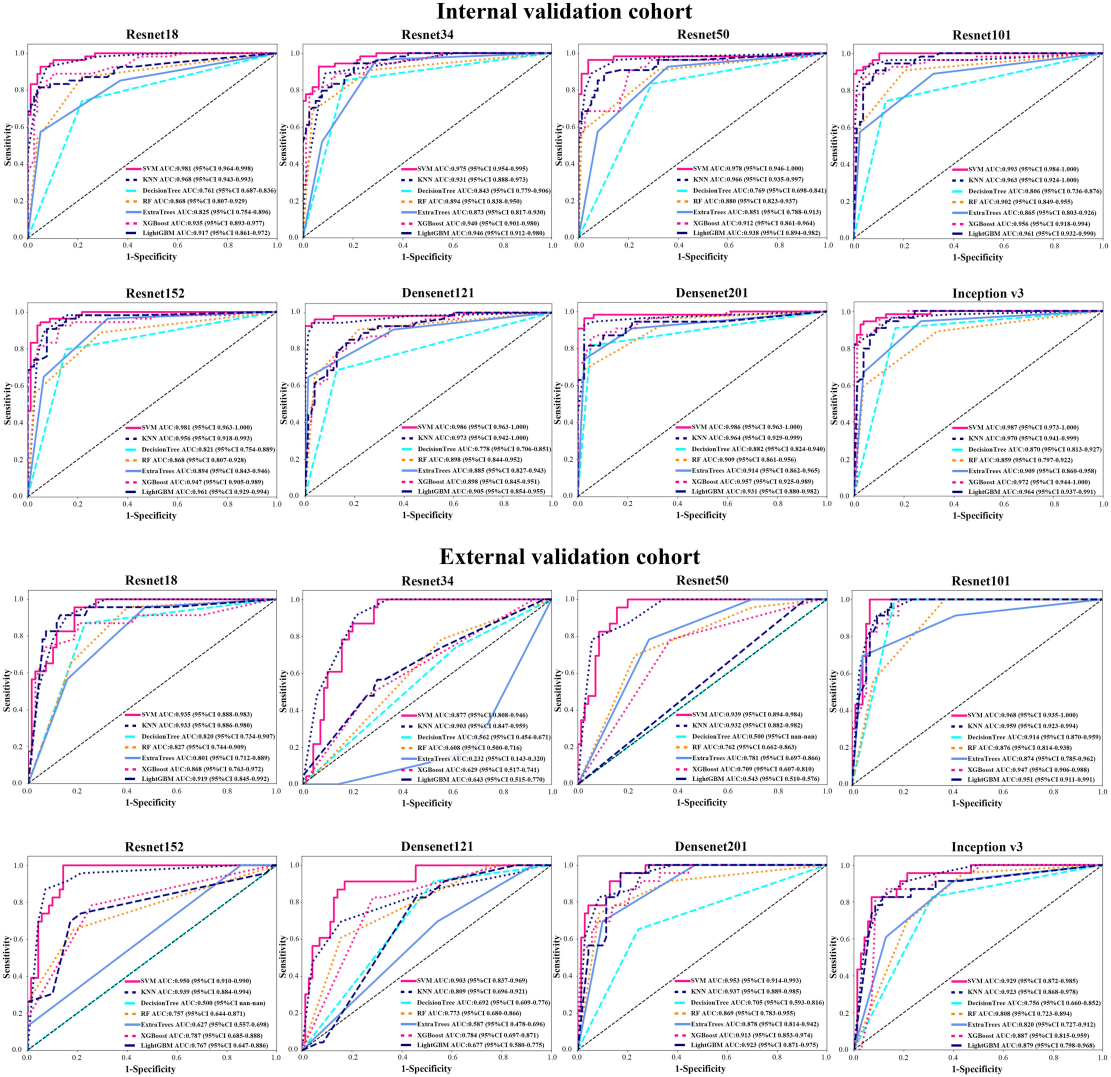
The AUC of various groups of deep learning feature (CNN) models in the internal and external validation cohorts using support vector machine (SVM), K-nearest neighbor (KNN), decision trees, random decision forests (RF), extra trees, XGBoost and lightGBM classifiers.

We analyzed the performance of ResNet18, ResNet34, ResNet50, ResNet101, ResNet152, Densenet121, Densenet201 and inception v3 to determine the best model for diagnosing EGC (**Table 2** and **Figure 3**). Our research showed that the ResNet101 model represented the best performance for diagnosing EGC with AUC 0.992 (95% CI: 0.984-1.000) and 0.968 (95% CI: 0.935-1.000) in the internal and external validation cohorts, respectively. Furthermore, the internal validation cohort had an accuracy of 93.1%, a sensitivity of 94.4%, and a specificity of 94.8%, meanwhile, the external validation cohort had an accuracy of 82.8%, a sensitivity of 100%, and a specificity of 85.7%.

**Table 2:** The difference between various deep learning models.

Models	Groups	AUC (95%CI)	Accuracy	Sensitivity	Specificity
**Resnet18**	Training	0.980 (0.969-0.991)	0.937	0.956	0.930
	Internal validation	0.981 (0.963-0.998)	0.924	0.925	0.948
	External validation	0.935 (0.888-0.983)	0.839	0.957	0.814
**Resnet34**	Training	0.979 (0.970-0.989)	0.916	0.995	0.873
	Internal validation	0.974 (0.954-0.995)	0.916	0.925	0.935
	External validation	0.877 (0.808-0.946)	0.796	1.000	0.710
**Resnet50**	Training	0.988 (0.981-0.994)	0.941	0.961	0.924
	Internal validation	0.977 (0.946-1.000)	0.962	0.962	0.961
	External validation	0.939 (0.894-0.984)	0.860	1.000	0.800
**Resnet101**	Training	0.975 (0.960-0.990)	0.946	0.966	0.949
	Internal validation	0.992 (0.984-1.000)	0.946	1.000	0.897
	External validation	0.968 (0.935-1.000)	0.914	1.000	0.929
**Resnet152**	Training	0.982 (0.971-0.992)	0.946	0.928	0.958
	Internal validation	0.981 (0.963-0.999)	0.931	0.944	0.948
	External validation	0.950 (0.911-0.990)	0.828	1.000	0.857
**Densenet121**	Training	0.996 (0.994-0.999)	0.965	0.995	0.952
	Internal validation	0.985 (0.963-1.000)	0.962	0.925	1.000
	External validation	0.903 (0.837-0.969)	0.806	0.913	0.829
**Densenet201**	Training	0.995 (0.992-0.998)	0.967	0.966	0.971
	Internal validation	0.985 (0.963-1.000)	0.969	0.962	0.974
	External validation	0.953 (0.914-0.993)	0.860	0.957	0.829
**Inception v3**	Training	0.986 (0.977-0.995)	0.944	0.961	0.949
	Internal validation	0.987 (0.973-1.000)	0.946	0.944	0.961
	External validation	0.929 (0.873-0.985)	0.839	0.826	0.929

AUC, area under the receiver operating characteristic curve; 95%CI, 95% confidence intervals.

**Figure 4:**
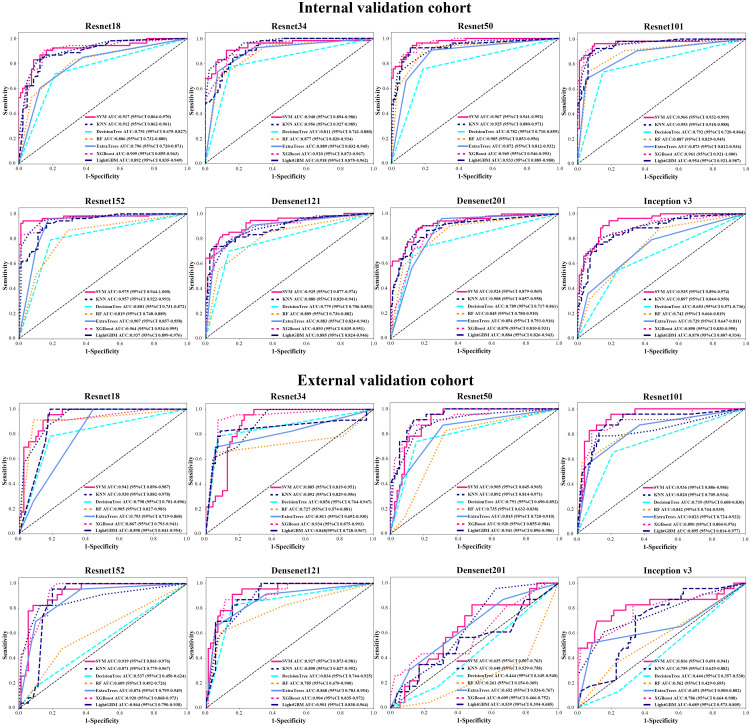
The AUC of various groups of deep transfer learning feature (pre-trained CNN) models in the internal and external validation cohorts using support vector machine (SVM), K-nearest neighbor (KNN), decision trees, random decision forests (RF), extra trees, XGBoost and lightGBM classifiers.

**Figure 5:**
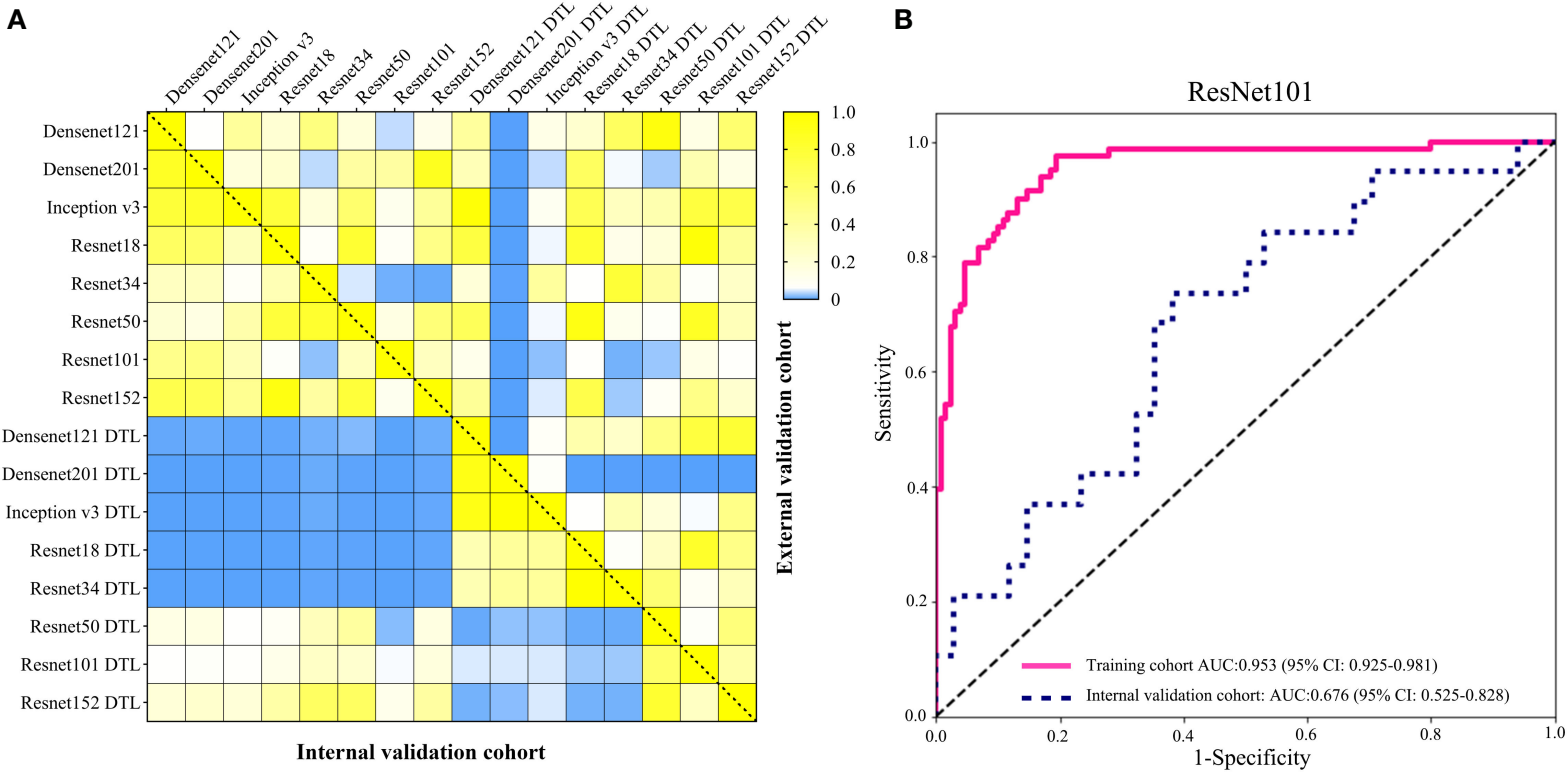
**(A)** The heatmap of the DeLong test *P*-value for various CNN and pre-trained CNN model of diagnosing EGC in the internal and external validation cohorts. The blue represented *P*-value < 0.05. **(B)** The AUC of ResNet101 model for diagnosing the depth of EGC in the training and internal validation cohort.

In order to make the CNN model more suitable for medical diagnosis scenarios, we pre-trained the various CNN models and constructed diagnosing EGC models. For the pre-trained model, in two validation cohorts, the pre-trained ResNet101 also represented better diagnosis ability with AUC 0.965 (95% CI: 0.932-0.999) and 0.936 (95% CI: 0.886-0.986) than others (**Table 3** and **Figure 4**). The internal validation cohort had an accuracy of 90.9%, a sensitivity of 96.2%, and a specificity of 89.8%, meanwhile, the external validation cohort had an accuracy of 89.2%, a sensitivity of 95.7%, and a specificity of 80.0%. Although the AUC score of several training cohorts showed better performance than validation cohorts, we thought the validation cohort was the most suitable data to evaluate the generalization ability of the model. **Figure 5A** represented the *P*-value of the DeLong test of different diagnosing EGC model in internal and external validation cohorts.

**Table 3:** The difference of various deep transfer learning models.

Models	Groups	AUC (95%CI)	Accuracy	Sensitivity	Specificity
**Resnet18**	Training	0.848 (0.814-0.882)	0.775	0.790	0.781
	Internal validation	0.917 (0.864-0.969)	0.825	0.830	0.911
	External validation	0.942 (0.900-0.987)	0.806	0.957	0.829
**Resnet34**	Training	0.886 (0.854-0.918)	0.823	0.914	0.765
	Internal validation	0.940 (0.894-0.985)	0.871	0.905	0.873
	External validation	0.885 (0.819-0.951)	0.785	0.957	0.771
**Resnet50**	Training	0.926 (0.904-0.949)	0.863	0.833	0.889
	Internal validation	0.966 (0.940-0.992)	0.886	0.943	0.873
	External validation	0.905 (0.845-0.965)	0.817	0.957	0.757
**Resnet101**	Training	0.904 (0.876-0.932)	0.849	0.833	0.870
	Internal validation	0.965 (0.932-0.999)	0.909	0.962	0.898
	External validation	0.936 (0.886-0.986)	0.892	0.957	0.800
**Resnet152**	Training	0.907 (0.881-0.934)	0.853	0.885	0.835
	Internal validation	0.975 (0.944-1.000)	0.954	0.943	0.974
	External validation	0.919 (0.861-0.977)	0.892	0.826	0.928
**Densenet121**	Training	0.930 (0.907-0.953)	0.853	0.933	0.819
	Internal validation	0.925 (0.877-0.974)	0.871	0.830	0.898
	External validation	0.927 (0.873-0.981)	0.860	0.913	0.843
**Densenet201**	Training	0.962 (0.948-0.977)	0.901	0.866	0.927
	Internal validation	0.924 (0.879-0.969)	0.833	0.886	0.835
	External validation	0.635 (0.506-0.763)	0.699	0.826	0.522
**Inception v3**	Training	0.953 (0.934-0.973)	0.882	0.923	0.901
	Internal validation	0.935 (0.895-0.974)	0.825	0.905	0.848
	External validation	0.816 (0.691-0.941)	0.806	0.696	0.886

AUC, area under the receiver operating characteristic curve; 95%CI, 95% confidence intervals.

### Performance of various machine learning classifications

We used different machine learning classifiers to develop diagnosing EGC models, including SVM, KNN, decision trees, RF, extra trees, XGBoost and lightGBM. In all classifiers, the AUC value of SVM classifier represented better performance than other classifiers both in CNN and pre-trained CNN models (**Supplementary Tables 3** and **4**). For example, in ResNet101 model, the AUCs value of the SVM, KNN, decision trees, RF, extra trees, XGBoost and lightGBM were 0.993 (95% CI: 0.985-1.000), 0.963 (95% CI: 0.924-1.000), 0.914 (95% CI: 0.870-0.959), 0.902 (95% CI: 0.850-0.955), 0.874 (95% CI: 0.785-0.962), 0.956 (95% CI: 0.918-0.994) and 0.956 (95% CI: 0.942-0.972) in the internal validation. Additionally, the accuracy of SVM classification was also better in terms of performance than KNN, decision trees, RF, extra trees, XGBoost and lightGBM classification, such as accuracy of 0.947, 0.939, 0.818, 0.826, 0.811, 0.682 and 0.738 in internal validation cohort and accuracy of 0.914, 0.903, 0.871, 0.828, 0.892, 0.839 and 0.903 in the external validation cohort.

## Discussion

With the advancement of human living conditions, the rise in the incidence of GC has drawn the attention of an increasing number of individuals. However, the majority of patients with GC received an advanced diagnosis after their initial diagnosis, which had a significant impact on their prognosis (23, 24). EGC has the characteristics of less treatment trauma and a 5-year overall survival rate of more than 90%, especially for endoscopic therapy was more acceptable to patients (2, 3). As a result, the diagnosis of EGC is particularly important. In recent years, AI technology has been widely used in the medical field, particularly in medical image analysis. The goal of this work is to use deep learning technology to create a CT diagnosis model of EGC and increase its efficiency. According to the Chinese recommendations for diagnosing and treating gastric cancer and the Japanese Gastric Cancer Association treatment guidelines, we can provide a more individualized diagnosis and treatment advice (5, 25).

To our knowledge, various researches were kept on diagnosing T staging of GC, including CT, EUS, double contrast-enhanced ultrasonography and laparoscopic exploration (6, 26–28). In enhanced CT portal vein phase images, the appearance of different densities in the normal stomach wall was used to discriminate T stage of GC (29). Radiologists diagnose T stage of GC based on the size of greatly enhanced lesions to the layers of the stomach wall in contrast-enhanced scans. The criterion of the T1 stage on CT image was as following: between the high enhancement of the inner part of the tumor and somewhat higher enhancement of the outside stomach muscle, there are continuous and complete low enhancement bands, or the highly enhanced lesions are not more than 50% of the total thickness of the stomach wall (25). Various previous studies showed that the accuracy of using CT to diagnose the T1 stage of GC was average from 63% to 82.7% and radiologists with different experiences had various rates to misdiagnose stage T1 as T2 (6, 30). Wang and colleagues reported a gastric window, which had a much higher accuracy more than 90% using CT diagnosing EGC (6). Although the strategy of adjusting the CT window can greatly enhance the diagnosis rate of EGC, this study had a small sample size, and the generalizability had to be confirmed further. In this study, the accuracy of the ResNet101 model diagnosing EGC was both 94.6% in the training and internal validation cohorts. In order to demonstrate the generalization performance of the CNN model, the external validation cohort that we collected also showed a good accuracy rate of 91.4%. To our surprise, the sensitivity of the diagnostic model in both validation cohorts reached 100%. The benefit of our findings is that we established a strategy to reduce the over-staging EGC patients, avoiding the need of unnecessary D2 lymphadenectomy or neoadjuvant treatment.

The depth of invasion of EGC is an essential evidence in deciding on endoscopic resection (31). Absolute indications for endoscopic resection of EGC, according to the second guidelines, are mostly for EGC with intramucosal invasion (5). Due to the high diagnostic rate of EUS, it is the first choice for evaluating the depth of invasion in EGC. Previous studies had explored the utility of CT in diagnosing the depth of invasion in EGC, but the final result was that EUS diagnosis was suggested before determining the treatment plan (6, 10). In this study, we tried to construct a model for identifying intramucosal EGC (T1a) using the ResNet101 neural network using the whole EGC patients from cohort 1. The results showed that the accuracy of diagnosing T1a was 88.6% and 62.3% in the training and internal validation cohorts (**Figure 5B**). The sensitivity of diagnosing T1a was 97.5% and 73.7%, and the specificity was 80.6% and 61.8% in the training and internal validation cohorts, respectively. In the internal validation cohort, the accuracy of diagnosing the T1a model was lower than Lee and Wang (6, 10). Compared to EUS diagnosing depth of EGC, the performance of CT images still faces a huge challenge.

In order to choose the best CNN model for diagnosing EGC, we developed various diagnostic models based on different neural network models, including ResNet18, ResNet34, ResNet50, ResNet101, ResNet152, Densenet121, Densenet201 and Inception v3 in this study. We found that the performance of CNN models for diagnosing EGC was almost no significant difference using the DeLong test. It justly seemed the AUC value of the ResNet101 model represented the highest compared to others. Deep learning model training needs a significant amount of processing power (32). When choosing the best model, the burden of a large computing load should also be considered, especially for big data in medical scenarios (33, 34). ResNet model uses residual learning to reduce gradient dispersion and accuracy loss in the deep networks, which can speed up the training of neural networks while improving model accuracy (35, 36). Resnet18 is an 18-layer convolutional neural network with fewer layers than ResNet101, and the result showed a satisfying ability of diagnosing EGC with AUC 0.981(95% CI: 0.963-0.998) and 0.935(95% CI: 0.888-0.983), and the accuracy of 92.4% and 83.9% in the internal and external validation cohorts. Densenets model significantly improves the transmission speed of information and gradients in the network, and it just requires half of the parameters and computation of ResNet to achieve the same accuracy (37, 38). The performance of the Densenet201 model for diagnosing EGC was also not inferior to the ResNet101 model with AUC 0.985(95% CI:0.963-1.000) and 0.953(95% CI: 0.914-0.993), and accuracy of 96.9% and 86.0% in the internal and external validation cohorts. In addition, previous studies indicated that pre-training CNN models with medical images to adjust the network parameters could significantly increase classification performance (39, 40). However, our results demonstrated that the pre-trained CNN model did not significantly improve its ability of diagnosing EGC, compared to the original CNN models, which may be due to insufficient training sample size. As a result, while treating clinical issues using a CNN model, not only the model’s performance but also the model’s applicability in hospitals must be addressed.

Artificial intelligence (AI) has advanced rapidly in biomedicine in recent years. The merging of AI technology and medical images such as endoscopy, radiographic images and pathology plays an important role in the diagnosis, staging and prognosis (41). Dong et al. employed a deep learning radiomics nomogram based on CNN to predict the degree of lymph node metastases in patients with advanced GC in an international multicenter trial (19). The model accurately distinguishes the degree of lymph node metastases in patients and is connected to the overall survival rate. Besides, Jiang et al. used CNN to build a model for predicting peritoneal metastasis of gastric cancer, and the AUC value exceeded 0.90, indicating a significant diagnostic benefit (20). Previous study indicates that AI technology has a good data analysis capability in the field of medical images; nevertheless, the low interpretability of AI is an important factor hindering its application (32, 41). Although some studies have improved their interpretability by examining the connection of deep learning features with clinical characteristics and radiomics features, they are still unable to match the present high demand for clinical proof (42, 43). In this study, we utilized deconvolution to create a heatmap of tumor images, but only explained the regions that the deep learning network focused on, without further explanation (**Supplementary Figure 4**). Therefore, the interpretability of deep learning features needs to be further explored to speed up its clinical application.

There are several limitations in this study (1). This study was a retrospective study, which is prone to sample selection bias. Therefore, follow-up prospective studies are needed to provide more clinical evidence (2). The patients included in this study were patients with confirmed GC diagnosis and lesions were found in the CT image. So this CNN model of diagnosing EGC did not applicable to EGC patients with no lesions found in CT images (3). We only used CT images of the portal vein phase to develop CNN diagnosing model in this study. In the future, we will employ several phases to construct the model.

## Conclusion

We firstly constructed a CNN prediction model for diagnosing EGC from GC patients, and the deep learning model also had the potential for differentiating between mucosa and submucosa tumors of EGC. These results suggest CNN model can provide favorable information for individualized treatment of EGC patients.

## Data availability statement

The original contributions presented in the study are included in the article/**Supplementary Material**. Further inquiries can be directed to the corresponding authors.

## Ethics statement

The studies involving human participants were reviewed and approved by The Ethics Committee of the First Affiliated Hospital of Nanchang University. Written informed consent for participation was not required for this study in accordance with the national legislation and the institutional requirements.

## Author contributions

QZ and ZF conceived the project and wrote the manuscript. YZhu and FZ drew the ROI of CT images. XS, AW, and LL participated in data analysis. YC and YT participated in the discussion and language editing. JX and ZL reviewed the manuscript. HL provided an external validation cohort. All authors contributed to the article and approved the submitted version.

## Funding

This work was supported by the National Natural Science Foundation of China (No.81860428), the leading scientists Project of Jiangxi Science and Technology Department (20213BCJL22050), and Youth Fund and Talent 555 Project of Jiangxi Provincial Science and Technology Department (20212BAB216036).

## Acknowledgments

We appreciate the Python technology offered by the OnekeyAI platform and thank the department of radiology for their support of CT images.

## Conflict of interest

The authors declare that the research was conducted in the absence of any commercial or financial relationships that could be construed as a potential conflict of interest.

## Publisher’s note

All claims expressed in this article are solely those of the authors and do not necessarily represent those of their affiliated organizations, or those of the publisher, the editors and the reviewers. Any product that may be evaluated in this article, or claim that may be made by its manufacturer, is not guaranteed or endorsed by the publisher.

## Supplementary material

Supplementary Figure 1 The inclusion criteria and exclusion criteria for the patients. EGC, early gastric cancer; CT, computed tomography; ESD, endoscopic submucosal dissection.

Supplementary Figure 3 Feature selection using LASSO logistic regression and the least absolute shrinkage. **(A)** Deep learning features selection; **(B)** Deep transfer learning features selection.

Supplementary Figure 4 The heatmap of the ResNet101 identified feature regions of diagnosing EGC models. The red region represented important.

Supplementary Table 1 Original deep learning features and deep transfer learning features.

Supplementary Table 2 Various models selected features of deep learning and deep transfer learning models.

Supplementary Table 4 The different performances of various classification models and the performance of various classifiers in different deep transfer learning (pre-trained CNN) models

## Abbreviations

EGC, early gastric cancer; DTL, deep transfer learning features; LASSO, the least absolute shrinkage and selection operator; SVM, support vector machine; KNN, K-Nearest Neighbor; RF, random decision forests; CT, computed tomography; CNNs, convolutional neural networks; ESD, endoscopic submucosal dissection; ROI, regions of interest; AI, artificial intelligence.
