# Supplementary material for: Deep learning model for diagnosing early gastric cancer using preoperative computed tomography images
Source: Front Oncol. 2022 Nov 30;12:1065934. doi: 10.3389/fonc.2022.1065934 (PMC9748811; doi:10.3389/fonc.2022.1065934)
Supplement: Supplementary Figure 1 — The inclusion criteria and exclusion criteria for the patients. EGC, early gastric cancer; CT, computed tomography; ESD, endoscopic submucosal dissection. [file DataSheet_1.zip › Figure S2.DOCX]

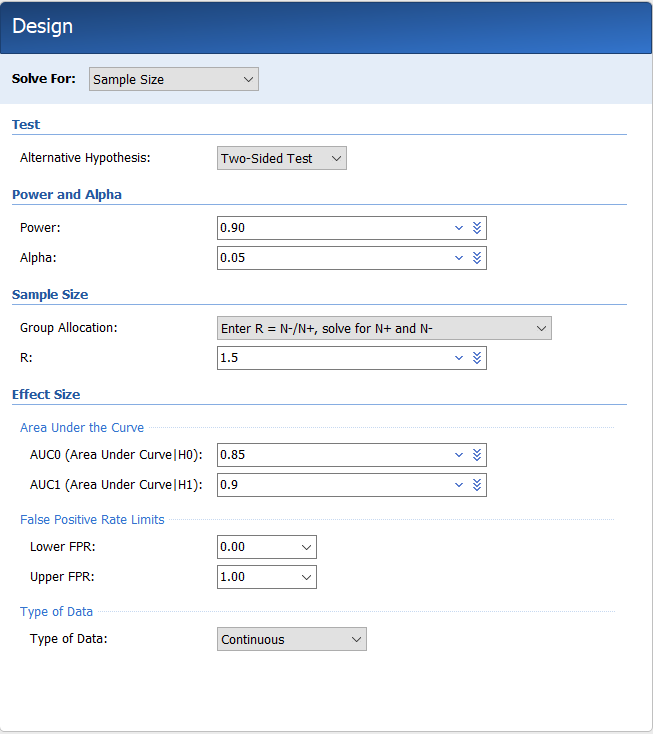

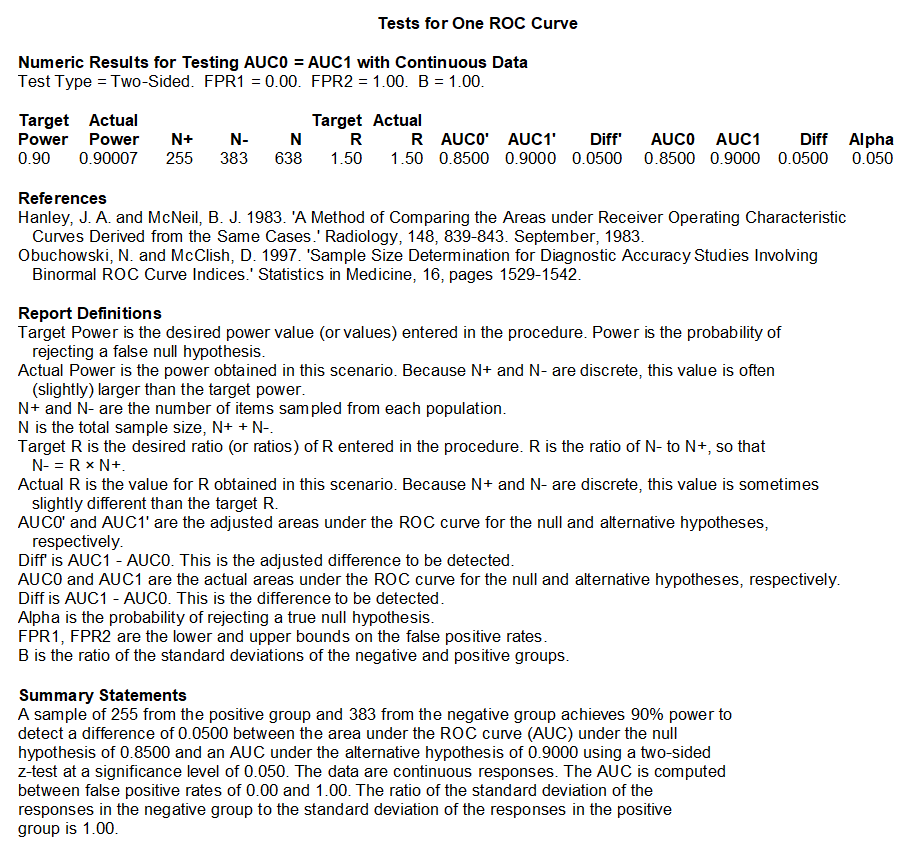


Figure S2 PASS sample size calculation of cohort1

A sample of 255 from the positive group and 383 from the negative group achieves 90% power to

detect a difference of 0.0500 between the area under the ROC curve (AUC) under the null hypothesis of 0.8500 and an AUC under the alternative hypothesis of 0.9000 using a two-sided z-test at a significance level of 0.050. The data are continuous responses. The AUC is computed between false positive rates of 0.00 and 1.00. The ratio of the standard deviation of the responses in the negative group to the standard deviation of the responses in the positive group is 1.00.
