# Supplementary material for: Deep learning model for diagnosing early gastric cancer using preoperative computed tomography images
Source: Front Oncol. 2022 Nov 30;12:1065934. doi: 10.3389/fonc.2022.1065934 (PMC9748811; doi:10.3389/fonc.2022.1065934)
Supplement: Supplementary Figure 1 — The inclusion criteria and exclusion criteria for the patients. EGC, early gastric cancer; CT, computed tomography; ESD, endoscopic submucosal dissection. [file DataSheet_1.zip › Figure S5.DOCX]

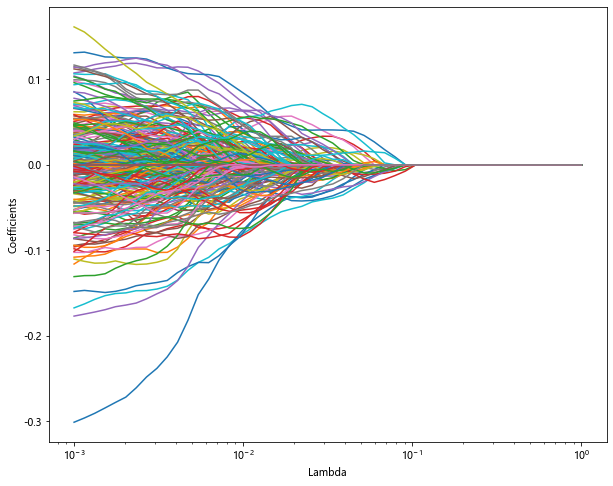

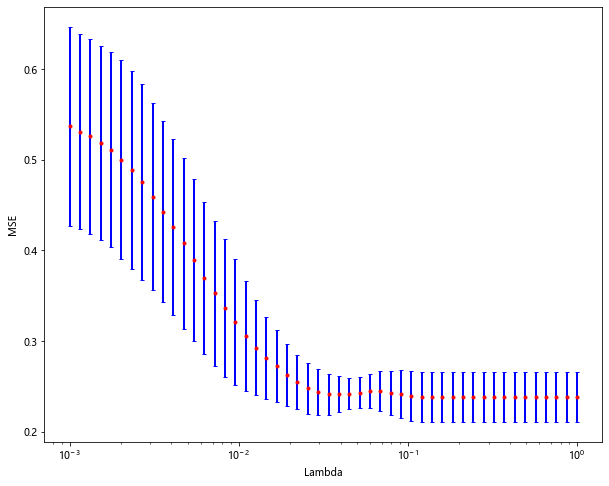
Supplementary Figure 5 the flowchart of the model distinguishing mucosa and submucosa tumors of EGC


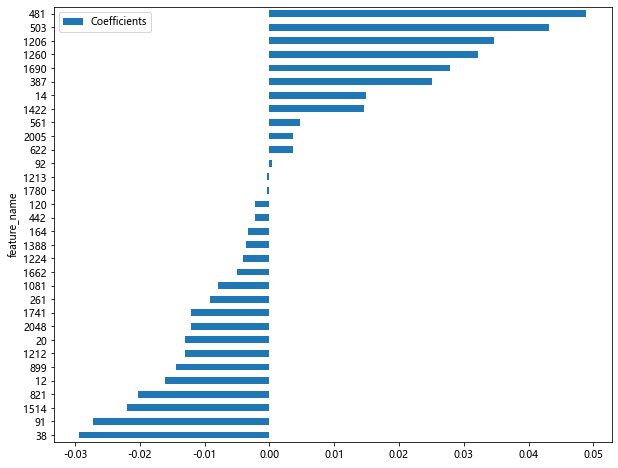
(A) Feature selection using LASSO logistic regression and the least absolute shrinkage.


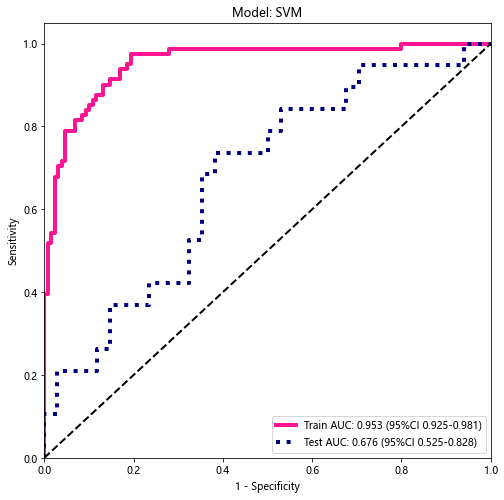
(B) The selected features of the diagnosing model

(C) The AUC of ResNet101 model for diagnosing the depth of EGC in the training and internal validation cohort.
