# Supplementary material for: Deep learning model for diagnosing early gastric cancer using preoperative computed tomography images
Source: Front Oncol. 2022 Nov 30;12:1065934. doi: 10.3389/fonc.2022.1065934 (PMC9748811; doi:10.3389/fonc.2022.1065934)
Supplement: Supplementary Figure 1 — The inclusion criteria and exclusion criteria for the patients. EGC, early gastric cancer; CT, computed tomography; ESD, endoscopic submucosal dissection. [file DataSheet_1.zip › Table S1.DOCX]

Table S1 The performance of various classifier in different deep learning models

| **Group** | **Classifier** | **Cohorts** | **AUC (95%CI)** | **Accuracy** | **Sensitivity** | **Specificity** |
| --- | --- | --- | --- | --- | --- | --- |
| **Resnet18** | SVM | Training | 0.980 (0.970-0.991) | 0.937 | 0.957 | 0.931 |
|  |  | Internal validation | 0.981 (0.964-0.998) | 0.924 | 0.926 | 0.949 |
|  |  | External validation | 0.935 (0.888-0.983) | 0.839 | 0.957 | 0.814 |
|  | KNN | Training | 0.948 (0.932-0.964) | 0.869 | 0.942 | 0.801 |
|  |  | Internal validation | 0.968 (0.943-0.993) | 0.924 | 0.926 | 0.923 |
|  |  | External validation | 0.933 (0.886-0.980) | 0.860 | 0.913 | 0.881 |
|  | DecisionTrees | Training | 1.000 | 1.000 | 1.000 | 1.000 |
|  |  | Internal validation | 0.761 (0.687-0.836) | 0.765 | 0.741 | 1.000 |
|  |  | External validation | 0.820 (0.734-0.907) | 0.796 | 0.870 | 1.000 |
|  | RF | Training | 0.970 (0.957-0.982) | 0.897 | 0.990 | 0.850 |
|  |  | Internal validation | 0.868 (0.807-0.929) | 0.788 | 0.870 | 0.813 |
|  |  | External validation | 0.827 (0.744-0.909) | 0.785 | 0.957 | 0.724 |
|  | ExtraTrees | Training | 1.000 | 1.000 | 1.000 | 1.000 |
|  |  | Internal validation | 0.825 (0.754-0.896) | 0.795 | 0.574 | 1.000 |
|  |  | External validation | 0.800 (0.712-0.889) | 0.774 | 0.957 | 0.627 |
|  | XGBoost | Training | 0.949 (0.933-0.966) | 0.736 | 0.823 | 0.918 |
|  |  | Internal validation | 0.935 (0.893-0.977) | 0.712 | 0.889 | 0.897 |
|  |  | External validation | 0.868 (0.763-0.972) | 0.828 | 0.870 | 0.800 |
|  | LightGBM | Training | 0.978 (0.966-0.992) | 0.937 | 0.938 | 0.949 |
|  |  | Internal validation | 0.917 (0.861-0.972) | 0.879 | 0.815 | 0.962 |
|  |  | External validation | 0.919 (0.845-0.992) | 0.828 | 0.913 | 0.924 |
| **Resnet34** | SVM | Training | 0.979 (0.970-0.989) | 0.916 | 0.995 | 0.874 |
|  |  | Internal validation | 0.975 (0.954-0.995) | 0.917 | 0.926 | 0.936 |
|  |  | External validation | 0.877 (0.808-0.946) | 0.796 | 1.000 | 0.710 |
|  | KNN | Training | 0.936 (0.917-0.955) | 0.857 | 0.847 | 0.864 |
|  |  | Internal validation | 0.931 (0.888-0.973) | 0.902 | 0.889 | 0.922 |
|  |  | External validation | 0.903 (0.847-0.959) | 0.742 | 0.913 | 0.833 |
|  | DecisionTrees | Training | 1.000 | 1.000 | 1.000 | 1.000 |
|  |  | Internal validation | 0.843 (0.780-0.906) | 0.841 | 0.852 | 1.000 |
|  |  | External validation | 0.562 (0.454-0.670) | 0.473 | 0.739 | 1.000 |
|  | RF | Training | 0.970 (0.957-0.983) | 0.907 | 0.987 | 0.867 |
|  |  | Internal validation | 0.894 (0.838-0.950) | 0.848 | 0.685 | 1.000 |
|  |  | External validation | 0.608 (0.499-0.716) | 0.731 | 0.783 | 0.463 |
|  | ExtraTrees | Training | 1.000 | 1.000 | 1.000 | 1.000 |
|  |  | Internal validation | 0.873 (0.817-0.930) | 0.758 | 0.963 | 0.764 |
|  |  | External validation | 0.232 (0.143-0.320) | 0.645 | 1.000 | 1.000 |
|  | XGBoost | Training | 0.957 (0.942-0.972) | 0.730 | 0.904 | 0.871 |
|  |  | Internal validation | 0.940 (0.900-0.980) | 0.697 | 0.889 | 0.872 |
|  |  | External validation | 0.629 (0.517-0.741) | 0.753 | 0.479 | 1.000 |
|  | LightGBM | Training | 0.989 (0.982-0.995) | 0.951 | 0.947 | 0.959 |
|  |  | Internal validation | 0.946 (0.912-0.980) | 0.856 | 0.944 | 0.795 |
|  |  | External validation | 0.643 (0.515-0.770) | 0.667 | 0.565 | 0.700 |
| **Resnet50** | SVM | Training | 0.988 (0.982-0.995) | 0.941 | 0.962 | 0.924 |
|  |  | Internal validation | 0.978 (0.947-1.000) | 0.962 | 0.963 | 0.962 |
|  |  | External validation | 0.939 (0.894-0.984) | 0.860 | 1.000 | 1.000 |
|  | KNN | Training | 0.957 (0.943-0.971) | 0.882 | 0.837 | 0.912 |
|  |  | Internal validation | 0.966 (0.935-0.997) | 0.924 | 0.889 | 0.949 |
|  |  | External validation | 0.932 (0.882-0.982) | 0.817 | 0.782 | 0.971 |
|  | DecisionTrees | Training | 1.000 | 1.000 | 1.000 | 1.000 |
|  |  | Internal validation | 0.769 (0.698-0.841) | 0.758 | 0.833 | 1.000 |
|  |  | External validation | 0.500 | 0.753 | 1.000 | 0 |
|  | RF | Training | 0.964 (0.950-0.980) | 0.875 | 0.976 | 0.882 |
|  |  | Internal validation | 0.880 (0.822-0.937) | 0.818 | 0.907 | 0.688 |
|  |  | External validation | 0.762 (0.662-0.863) | 0.753 | 0.696 | 1.000 |
|  | ExtraTrees | Training | 1.000 | 1.000 | 1.000 | 1.000 |
|  |  | Internal validation | 0.851 (0.788-0.913) | 0.780 | 0.926 | 0.694 |
|  |  | External validation | 0.781 (0.697-0.866) | 0.731 | 0.783 | 1.000 |
|  | XGBoost | Training | 0.947 (0.931-0.963) | 0.772 | 0.952 | 0.785 |
|  |  | Internal validation | 0.912 (0.861-0.964) | 0.788 | 0.907 | 0.795 |
|  |  | External validation | 0.709 (0.607-0.810) | 0.753 | 0.783 | 1.000 |
|  | LightGBM | Training | 0.982 (0.970-0.994) | 0.941 | 0.962 | 0.946 |
|  |  | Internal validation | 0.938 (0.894-0983) | 0.879 | 0.889 | 0.897 |
|  |  | External validation | 0.543 (0.510-0.576) | 0.312 | 1.000 | 1.000 |
| **Resnet101** | SVM | Training | 0.975 (0.960-0.990) | 0.947 | 0.967 | 0.950 |
|  |  | Internal validation | 0.993 (0.985-1.000) | 0.947 | 1.000 | 0.897 |
|  |  | External validation | 0.968 (0.935-1.000) | 0.914 | 1.000 | 0.929 |
|  | KNN | Training | 0.959 (0.945-0.973) | 0.894 | 0.914 | 0.855 |
|  |  | Internal validation | 0.963 (0.924-1.000) | 0.939 | 0.926 | 0.949 |
|  |  | External validation | 0.959 (0.923-0.994) | 0.903 | 0.913 | 0.926 |
|  | DecisionTrees | Training | 1.000 | 1.000 | 1.000 | 1.000 |
|  |  | Internal validation | 0.806 (0.737-0.876) | 0.818 | 0.741 | 1.000 |
|  |  | External validation | 0.914 (0.870-0.959) | 0.871 | 1.000 | 1.000 |
|  | RF | Training | 0.971 (0.957-0.984) | 0.913 | 0.981 | 0.860 |
|  |  | Internal validation | 0.902 (0.850-0.955) | 0.826 | 0.907 | 0.816 |
|  |  | External validation | 0.876 (0.815-0.938) | 0.828 | 1.000 | 0.688 |
|  | ExtraTrees | Training | 1.000 | 1.000 | 1.000 | 1.000 |
|  |  | Internal validation | 0.865 (0.803-0.926) | 0.811 | 0.889 | 0.697 |
|  |  | External validation | 0.874 (0.785-0.962) | 0.892 | 0.696 | 1.000 |
|  | XGBoost | Training | 0.957 (0.942-0.972) | 0.738 | 0.866 | 0.909 |
|  |  | Internal validation | 0.956 (0.918-0.994) | 0.682 | 0.870 | 0.962 |
|  |  | External validation | 0.947 (0.906-0.989) | 0.839 | 1.000 | 0.814 |
|  | LightGBM | Training | 0.989 (0.982-0.996) | 0.947 | 0.971 | 0.946 |
|  |  | Internal validation | 0.956 (0.942-0.972) | 0.738 | 0.870 | 0.962 |
|  |  | External validation | 0.951 (0.911-0.991) | 0.903 | 1.000 | 0.868 |
| **Resnet152** | SVM | Training | 0.982 (0.971-0.993) | 0.947 | 0.928 | 0.959 |
|  |  | Internal validation | 0.981 (0.963-1.000) | 0.932 | 0.944 | 0.949 |
|  |  | External validation | 0.950 (0.910-0.990) | 0.828 | 1.000 | 0.857 |
|  | KNN | Training | 0.955 (0.940-0.970) | 0.894 | 0.847 | 0.924 |
|  |  | Internal validation | 0.956 (0.918-0.993) | 0.917 | 0.981 | 0.868 |
|  |  | External validation | 0.939 (0.884-0.994) | 0.860 | 0.870 | 0.970 |
|  | DecisionTrees | Training | 1.000 | 1.000 | 1.000 | 1.000 |
|  |  | Internal validation | 0.821 (0.754-0.889) | 0.826 | 0.796 | 1.000 |
|  |  | External validation | 0.500 | 0.753 | 1.000 | 0 |
|  | RF | Training | 0.968 (0.954-0.983) | 0.913 | 0.976 | 0.863 |
|  |  | Internal validation | 0.868 (0.807-0.928) | 0.803 | 0.889 | 0.724 |
|  |  | External validation | 0.757 (0.644-0.871) | 0.817 | 0.652 | 0.814 |
|  | ExtraTrees | Training | 1.000 | 1.000 | 1.000 | 1.000 |
|  |  | Internal validation | 0.894 (0.843-0.946) | 0.818 | 0.963 | 0.726 |
|  |  | External validation | 0.627 (0.557-0.698) | 0.785 | 1.000 | 0.143 |
|  | XGBoost | Training | 0.951 (0.936-0.967) | 0.776 | 0.904 | 0.836 |
|  |  | Internal validation | 0.947 (0.905-0.989) | 0.811 | 0.926 | 0.872 |
|  |  | External validation | 0.787 (0.685-0.889) | 0.753 | 0.783 | 0.743 |
|  | LightGBM | Training | 0.984 (0.973-0.995) | 0.952 | 0.947 | 0.959 |
|  |  | Internal validation | 0.961 (0.929-0.994) | 0.879 | 0.907 | 0.923 |
|  |  | External validation | 0.767 (0.648-0.886) | 0.774 | 0.739 | 0.786 |
| **Densenet121** | SVM | Training | 0.997 (0.995-0.999) | 0.966 | 0.995 | 0.953 |
|  |  | Internal validation | 0.986 (0.963-1.000) | 0.963 | 0.926 | 1.000 |
|  |  | External validation | 0.903 (0.837-0.969) | 0.806 | 0.913 | 0.829 |
|  | KNN | Training | 0.978 (0.969-0.987) | 0.924 | 0.923 | 0.924 |
|  |  | Internal validation | 0.973 (0.942-1.000) | 0.955 | 0.944 | 0.962 |
|  |  | External validation | 0.809 (0.696-0.921) | 0.570 | 0.696 | 0.881 |
|  | DecisionTrees | Training | 1.000 | 1.000 | 1.000 | 1.000 |
|  |  | Internal validation | 0.778 (0.706-0.851) | 0.795 | 0.685 | 1.000 |
|  |  | External validation | 0.692 (0.609-0.776) | 0.581 | 0.913 | 1.000 |
|  | RF | Training | 0.962 (0.947-0.978) | 0.897 | 0.976 | 0.859 |
|  |  | Internal validation | 0.898 (0.844-0.952) | 0.833 | 0.907 | 0.813 |
|  |  | External validation | 0.773 (0.681-0.866) | 0.785 | 0.609 | 1.000 |
|  | ExtraTrees | Training | 1.000 | 1.000 | 1.000 | 1.000 |
|  |  | Internal validation | 0.885 (0.827-0.943) | 0.848 | 0.648 | 1.000 |
|  |  | External validation | 0.587 (0.478-0.696) | 0.516 | 0.696 | 1.000 |
|  | XGBoost | Training | 0.898 (0.845-0.951) | 0.704 | 0.833 | 0.833 |
|  |  | Internal validation | 0.963 (0.949-0.976) | 0.730 | 0.876 | 0.902 |
|  |  | External validation | 0.784 (0.697-0.871) | 0.753 | 0.826 | 1.000 |
|  | LightGBM | Training | 0.995 (0.989-0.999) | 0.964 | 0.967 | 0.978 |
|  |  | Internal validation | 0.905 (0.854-0.955) | 0.811 | 0.852 | 0.831 |
|  |  | External validation | 0.677 (0.580-0.775) | 0.613 | 0.826 | 0.585 |
| **Densenet201** | SVM | Training | 0.995 (0.992-0.999) | 0.968 | 0.967 | 0.972 |
|  |  | Internal validation | 0.986 (0.963-1.000) | 0.970 | 0.963 | 0.974 |
|  |  | External validation | 0.953 (0.914-0.993) | 0.860 | 0.957 | 0.829 |
|  | KNN | Training | 0.970 (0.959-0.981) | 0.903 | 0.933 | 0.874 |
|  |  | Internal validation | 0.964 (0.929-0.999) | 0.955 | 0.926 | 0.974 |
|  |  | External validation | 0.937 (0.889-0.985) | 0.796 | 0.826 | 0.941 |
|  | DecisionTrees | Training | 1.000 | 1.000 | 1.000 | 1.000 |
|  |  | Internal validation | 0.882 (0.824-0.940) | 0.894 | 0.815 | 1.000 |
|  |  | External validation | 0.705 (0.593-0.816) | 0.731 | 0.652 | 1.000 |
|  | RF | Training | 0.975 (0.963-0.987) | 0.913 | 0.981 | 0.873 |
|  |  | Internal validation | 0.909 (0.861-0.956) | 0.856 | 0.685 | 1.000 |
|  |  | External validation | 0.869 (0.783-0.955) | 0.871 | 0.739 | 1.000 |
|  | ExtraTrees | Training | 1.000 | 1.000 | 1.000 | 1.000 |
|  |  | Internal validation | 0.914 (0.862-0.966) | 0.879 | 0.741 | 1.000 |
|  |  | External validation | 0.878 (0.814-0.942) | 0.849 | 0.696 | 1.000 |
|  | XGBoost | Training | 0.977 (0.967-0.987) | 0.787 | 0.957 | 0.880 |
|  |  | Internal validation | 0.957 (0.925-0.989) | 0.826 | 0.852 | 0.949 |
|  |  | External validation | 0.913 (0.853-0.974) | 0.860 | 0.913 | 0.743 |
|  | LightGBM | Training | 0.992 (0.984-0.999) | 0.960 | 0.962 | 0.965 |
|  |  | Internal validation | 0.931 (0.880-0.983) | 0.879 | 0.815 | 0.974 |
|  |  | External validation | 0.923 (0.871-0.975) | 0.860 | 0.957 | 0.866 |
| **Inception v3** | SVM | Training | 0.986 (0.977-0.996) | 0.945 | 0.962 | 0.950 |
|  |  | Internal validation | 0.987 (0.973-1.000) | 0.947 | 0.944 | 0.962 |
|  |  | External validation | 0.929 (0.873-0.985) | 0.839 | 0.826 | 0.929 |
|  | KNN | Training | 0.951 (0.936-0.967) | 0.878 | 0.913 | 0.845 |
|  |  | Internal validation | 0.970 (0.941-0.999) | 0.909 | 0.815 | 1.000 |
|  |  | External validation | 0.923 (0.868-0.978) | 0.817 | 0.826 | 0.925 |
|  | DecisionTrees | Training | 1.000 | 1.000 | 1.000 | 1.000 |
|  |  | Internal validation | 0.870 (0.813-0.927) | 0.864 | 0.907 | 1.000 |
|  |  | External validation | 0.756 (0.660-0.852) | 0.720 | 0.826 | 1.000 |
|  | RF | Training | 0.962 (0.946-0.977) | 0.897 | 0.976 | 0.868 |
|  |  | Internal validation | 0.859 (0.797-0.922) | 0.811 | 0.889 | 0.693 |
|  |  | External validation | 0.808 (0.723-0.894) | 0.763 | 0.957 | 0.709 |
|  | ExtraTrees | Training | 1.000 | 1.000 | 1.000 | 1.000 |
|  |  | Internal validation | 0.909 (0.860-0.958) | 0.841 | 0.944 | 0.760 |
|  |  | External validation | 0.820 (0.728-0.912) | 0.806 | 0.913 | 0.689 |
|  | XGBoost | Training | 0.952 (0.936-0.967) | 0.741 | 0.923 | 0.817 |
|  |  | Internal validation | 0.972 (0.944-0.999) | 0.758 | 0.963 | 0.898 |
|  |  | External validation | 0.887 (0.815-0.960) | 0.892 | 0.870 | 0.897 |
|  | LightGBM | Training | 0.979 (0.966-0.993) | 0.951 | 0.937 | 0.965 |
|  |  | Internal validation | 0.964 (0.937-0.992) | 0.902 | 0.907 | 0.922 |
|  |  | External validation | 0.879 (0.789-0.968) | 0.785 | 0.826 | 0.954 |
