# Supplementary material for: Deep learning model for diagnosing early gastric cancer using preoperative computed tomography images
Source: Front Oncol. 2022 Nov 30;12:1065934. doi: 10.3389/fonc.2022.1065934 (PMC9748811; doi:10.3389/fonc.2022.1065934)
Supplement: Supplementary Figure 1 — The inclusion criteria and exclusion criteria for the patients. EGC, early gastric cancer; CT, computed tomography; ESD, endoscopic submucosal dissection. [file DataSheet_1.zip › Table S2.DOCX]

Table S2 The performance of various classifier in different deep transfer learning models

| **Group** | **Classifier** | **Cohorts** | **AUC (95%CI)** | **Accuracy** | **Sensitivity** | **Specificity** |
| --- | --- | --- | --- | --- | --- | --- |
| **Resnet18** | SVM | Training | 0.849 (0.815-0.883) | 0.776 | 0.790 | 0.782 |
|  |  | Internal validation | 0.917 (0.865-0.970) | 0.826 | 0.830 | 0.911 |
|  |  | External validation | 0.941 (0.896-0.987) | 0.806 | 0.957 | 0.829 |
|  | KNN | Training | 0.904 (0.881-0.928) | 0.833 | 0.767 | 0.877 |
|  |  | Internal validation | 0.912 (0.862-0.961) | 0.864 | 0.830 | 0.886 |
|  |  | External validation | 0.930 (0.882-0.978) | 0.806 | 1.000 | 0.721 |
|  | DecisionTrees | Training | 1.000 | 1.000 | 1.000 | 1.000 |
|  |  | Internal validation | 0.751 (0.675-0.827) | 0.758 | 0.717 | 1.000 |
|  |  | External validation | 0.798 (0.701-0.896) | 0.806 | 0.783 | 1.000 |
|  | RF | Training | 0.957 (0.941-0.974) | 0.895 | 0.971 | 0.839 |
|  |  | Internal validation | 0.806 (0.732-0.880) | 0.765 | 0.830 | 0.726 |
|  |  | External validation | 0.905 (0.827-0.983) | 0.914 | 0.913 | 1.000 |
|  | ExtraTrees | Training | 1.000 | 1.000 | 1.000 | 1.000 |
|  |  | Internal validation | 0.796 (0.720-0.871) | 0.765 | 0.604 | 1.000 |
|  |  | External validation | 0.793 (0.719-0.868) | 0.753 | 1.000 | 0.609 |
|  | XGBoost | Training | 0.900 (0.875-0.925) | 0.667 | 0.905 | 0.725 |
|  |  | Internal validation | 0.909 (0.855-0.963) | 0.697 | 0.906 | 0.848 |
|  |  | External validation | 0.867 (0.793-0.941) | 0.753 | 0.913 | 0.983 |
|  | LightGBM | Training | 0.928 (0.904-0.952) | 0.876 | 0.833 | 0.911 |
|  |  | Internal validation | 0.892 (0.835-0.949) | 0.818 | 0.868 | 0.870 |
|  |  | External validation | 0.898 (0.841-0.954) | 0.849 | 1.000 | 0.966 |
| **Resnet34** | SVM | Training | 0.886 (0.854-0.918) | 0.823 | 0.914 | 0.766 |
|  |  | Internal validation | 0.940 (0.894-0.986) | 0.871 | 0.906 | 0.873 |
|  |  | External validation | 0.849 (0.815-0.883) | 0.776 | 0.790 | 0.782 |
|  | KNN | Training | 0.929 (0.910-0.948) | 0.840 | 0.914 | 0.766 |
|  |  | Internal validation | 0.956 (0.927-0.985) | 0.856 | 0.962 | 0.772 |
|  |  | External validation | 0.892 (0.829-0.956) | 0.785 | 0.767 | 0.877 |
|  | DecisionTrees | Training | 1.000 | 1.000 | 1.000 | 1.000 |
|  |  | Internal validation | 0.811 (0.741-0.880) | 0.818 | 0.774 | 1.000 |
|  |  | External validation | 0.856 (0.764-0.947) | 0.892 | 0.783 | 1.000 |
|  | RF | Training | 0.960 (0.944-0.976) | 0.892 | 0.976 | 0.871 |
|  |  | Internal validation | 0.877 (0.820-0.934) | 0.826 | 0.680 | 1.000 |
|  |  | External validation | 0.727 (0.574-0.881) | 0.871 | 0.652 | 1.000 |
|  | ExtraTrees | Training | 1.000 | 1.000 | 1.000 | 1.000 |
|  |  | Internal validation | 0.889 (0.832-0.945) | 0.848 | 0.736 | 1.000 |
|  |  | External validation | 0.811 (0.692-0.930) | 0.882 | 0.696 | 1.000 |
|  | XGBoost | Training | 0.935 (0.916-0.954) | 0.753 | 0.833 | 0.858 |
|  |  | Internal validation | 0.920 (0.873-0.967) | 0.788 | 0.868 | 0.848 |
|  |  | External validation | 0.934 (0.875-0.993) | 0.753 | 0.913 | 0.970 |
|  | LightGBM | Training | 0.967 (0.951-0.983) | 0.916 | 0.952 | 0.901 |
|  |  | Internal validation | 0.918 (0.875-0.962) | 0.841 | 0.849 | 0.835 |
|  |  | External validation | 0.848 (0.728-0.967) | 0.882 | 0.826 | 0.955 |
| **Resnet50** | SVM | Training | 0.927 (0.904-0.950) | 0.863 | 0.833 | 0.889 |
|  |  | Internal validation | 0.967 (0.941-0.992) | 0.886 | 0.943 | 0.873 |
|  |  | External validation | 0.885 (0.819-0.951) | 0.785 | 0.957 | 0.771 |
|  | KNN | Training | 0.938 (0.920-0.956) | 0.867 | 0.810 | 0.905 |
|  |  | Internal validation | 0.925 (0.880-0.971) | 0.848 | 0.811 | 0.885 |
|  |  | External validation | 0.892 (0.829-0.956) | 0.785 | 1.000 | 0.657 |
|  | DecisionTrees | Training | 1.000 | 1.000 | 1.000 | 1.000 |
|  |  | Internal validation | 0.782 (0.710-0.855) | 0.788 | 0.755 | 1.000 |
|  |  | External validation | 0.856 (0.764-0.947) | 0.892 | 0.783 | 1.000 |
|  | RF | Training | 0.970 (0.956-0.983) | 0.907 | 0.986 | 0.881 |
|  |  | Internal validation | 0.905 (0.853-0.956) | 0.818 | 0.925 | 0.829 |
|  |  | External validation | 0.727 (0.574-0.881) | 0.871 | 0.652 | 1.000 |
|  | ExtraTrees | Training | 1.000 | 1.000 | 1.000 | 1.000 |
|  |  | Internal validation | 0.872 (0.812-0.932) | 0.811 | 0.906 | 0.833 |
|  |  | External validation | 0.811 (0.692-0.930) | 0.882 | 0.696 | 1.000 |
|  | XGBoost | Training | 0.959 (0.945-0.974) | 0.730 | 0.900 | 0.892 |
|  |  | Internal validation | 0.969 (0.946-0.993) | 0.727 | 0.868 | 0.937 |
|  |  | External validation | 0.934 (0.875-0.993) | 0.753 | 0.913 | 0.970 |
|  | LightGBM | Training | 0.977 (0.964-0.991) | 0.951 | 0.924 | 0.968 |
|  |  | Internal validation | 0.933 (0.885-0.980) | 0.879 | 0.925 | 0.886 |
|  |  | External validation | 0.848 (0.728-0.967) | 0.882 | 0.826 | 0.955 |
| **Resnet101** | SVM | Training | 0.905 (0.877-0.933) | 0.850 | 0.833 | 0.870 |
|  |  | Internal validation | 0.966 (0.932-0.999) | 0.909 | 0.962 | 0.899 |
|  |  | External validation | 0.936 (0.886-0.986) | 0.892 | 0.957 | 0.800 |
|  | KNN | Training | 0.940 (0.923-0.958) | 0.857 | 0.919 | 0.785 |
|  |  | Internal validation | 0.953 (0.918-0.988) | 0.901 | 0.849 | 0.937 |
|  |  | External validation | 0.820 (0.705-0.935) | 0.860 | 0.783 | 0.899 |
|  | DecisionTrees | Training | 1.000 | 1.000 | 1.000 | 1.000 |
|  |  | Internal validation | 0.792 (0.720-0.864) | 0.803 | 0.736 | 1.000 |
|  |  | External validation | 0.719 (0.608-0.830) | 0.753 | 0.652 | 1.000 |
|  | RF | Training | 0.961 (0.946-0.977) | 0.903 | 0.976 | 0.852 |
|  |  | Internal validation | 0.887 (0.829-0.946) | 0.856 | 0.736 | 1.000 |
|  |  | External validation | 0.842 (0.744-0.939) | 0.871 | 0.565 | 1.000 |
|  | ExtraTrees | Training | 1.000 | 1.000 | 1.000 | 1.000 |
|  |  | Internal validation | 0.873 (0.812-0.934) | 0.841 | 0.679 | 1.000 |
|  |  | External validation | 0.823 (0.724-0.922) | 0.839 | 0.870 | 0.677 |
|  | XGBoost | Training | 0.942 (0.924-0.960) | 0.686 | 0.814 | 0.899 |
|  |  | Internal validation | 0.961 (0.921-1.000) | 0.750 | 0.962 | 0.899 |
|  |  | External validation | 0.890 (0.804-0.976) | 0.686 | 0.814 | 0.899 |
|  | LightGBM | Training | 0.966 (0.951-0.981) | 0.907 | 0.933 | 0.898 |
|  |  | Internal validation | 0.954 (0.921-0.987) | 0.902 | 0.925 | 0.899 |
|  |  | External validation |  |  |  |  |
| **Resnet152** | SVM | Training | 0.908 (0.881-0.934) | 0.854 | 0.886 | 0.835 |
|  |  | Internal validation | 0.975 (0.944-1.000) | 0.955 | 0.943 | 0.975 |
|  |  | External validation | 0.919 (0.861-0.977) | 0.892 | 0.826 | 0.928 |
|  | KNN | Training | 0.940 (0.922-0.957) | 0.857 | 0.933 | 0.769 |
|  |  | Internal validation | 0.957 (0.922-0.995) | 0.902 | 0.943 | 0.872 |
|  |  | External validation | 0.871 (0.776-0.967) | 0.860 | 0.826 | 0.884 |
|  | DecisionTrees | Training | 1.000 | 1.000 | 1.000 | 1.000 |
|  |  | Internal validation | 0.801 (0.731-0.872) | 0.803 | 0.792 | 1.000 |
|  |  | External validation | 0.537 (0.450-0.624) | 0.720 | 0.174 | 1.000 |
|  | RF | Training | 0.970 (0.956-0.984) | 0.924 | 0.976 | 0.869 |
|  |  | Internal validation | 0.819 (0.748-0.889) | 0.742 | 0.868 | 0.789 |
|  |  | External validation | 0.609 (0.492-0.726) | 0.742 | 0.478 | 0.765 |
|  | ExtraTrees | Training | 1.000 | 1.000 | 1.000 | 1.000 |
|  |  | Internal validation | 0.907 (0.857-0.958) | 0.826 | 0.962 | 0.915 |
|  |  | External validation | 0.874 (0.799-0.949) | 0.849 | 0.696 | 1.000 |
|  | XGBoost | Training | 0.955 (0.940-0.970) | 0.764 | 0.929 | 0.823 |
|  |  | Internal validation | 0.964 (0.934-0.995) | 0.780 | 0.830 | 0.987 |
|  |  | External validation | 0.920 (0.868-0.973) | 0.839 | 1.000 | 0.783 |
|  | LightGBM | Training | 0.980 (0.969-0.991) | 0.935 | 0.952 | 0.934 |
|  |  | Internal validation | 0.937 (0.899-0.976) | 0.871 | 0.925 | 0.835 |
|  |  | External validation | 0.920 (0.868-0.973) | 0.839 | 1.000 | 0.783 |
| **Densenet121** | SVM | Training | 0.930 (0.907-0.954) | 0.854 | 0.933 | 0.820 |
|  |  | Internal validation | 0.925 (0.877-0.974) | 0.871 | 0.830 | 0.899 |
|  |  | External validation | 0.927 (0.873-0.981) | 0.860 | 0.913 | 0.843 |
|  | KNN | Training | 0.912 (0.890-0.935) | 0.840 | 0.752 | 0.899 |
|  |  | Internal validation | 0.880 (0.820-0.941) | 0.833 | 0.774 | 0.885 |
|  |  | External validation | 0.890 (0.827-0.952) | 0.763 | 1.000 | 0.691 |
|  | DecisionTrees | Training | 1.000 | 1.000 | 1.000 | 1.000 |
|  |  | Internal validation | 0.779 (0.706-0.853) | 0.795 | 0.698 | 1.000 |
|  |  | External validation | 0.834 (0.744-0.925) | 0.839 | 0.826 | 1.000 |
|  | RF | Training | 0.965 (0.951-0.980) | 0.911 | 0.976 | 0.822 |
|  |  | Internal validation | 0.809 (0.736-0.882) | 0.773 | 0.849 | 0.685 |
|  |  | External validation | 0.785 (0.670-0.900) | 0.839 | 0.609 | 1.000 |
|  | ExtraTrees | Training | 1.0000 | 1.000 | 1.000 | 1.000 |
|  |  | Internal validation | 0.883 (0.824-0.941) | 0.8333 | 0.906 | 0.760 |
|  |  | External validation | 0.868 (0.783-0.954) | 0.871 | 0.652 | 1.000 |
|  | XGBoost | Training | 0.951 (0.935-0.968) | 0.734 | 0.890 | 0.877 |
|  |  | Internal validation | 0.893 (0.835-0.951) | 0.742 | 0.811 | 0.848 |
|  |  | External validation | 0.904 (0.835-0.972) | 0.753 | 0.870 | 0.899 |
|  | LightGBM | Training | 0.979 (0.966-0.991) | 0.947 | 0.938 | 0.956 |
|  |  | Internal validation | 0.885 (0.824-0.946) | 0.848 | 0.736 | 0.949 |
|  |  | External validation | 0.900 (0.838-0.964) | 0.806 | 0.870 | 0.814 |
| **Densenet201** | SVM | Training | 0.963 (0.948-0.977) | 0.901 | 0.867 | 0.927 |
|  |  | Internal validation | 0.924 (0.879-0.970) | 0.833 | 0.887 | 0.835 |
|  |  | External validation | 0.635 (0.507-0.763) | 0.699 | 0.826 | 0.522 |
|  | KNN | Training | 0.921 (0.900-0.942) | 0.844 | 0.814 | 0.864 |
|  |  | Internal validation | 0.908 (0.857-0.959) | 0.848 | 0.906 | 0.821 |
|  |  | External validation | 0.648 (0.539-0.758) | 0.613 | 0.957 | 1.000 |
|  | DecisionTrees | Training | 1.000 | 1.000 | 1.000 | 1.000 |
|  |  | Internal validation | 0.789 (0.717-0.861) | 0.803 | 0.717 | 1.000 |
|  |  | External validation | 0.444 (0.349-0.540) | 0.581 | 1.000 | 1.000 |
|  | RF | Training | 0.967 (0.952-0.981) | 0.916 | 0.981 | 0.839 |
|  |  | Internal validation | 0.845 (0.780-0.910) | 0.788 | 0.906 | 0.699 |
|  |  | External validation | 0.261 (0.154-0.370) | 0.591 | 1.000 | 1.000 |
|  | ExtraTrees | Training | 1.000 | 1.000 | 1.000 | 1.000 |
|  |  | Internal validation | 0.854 (0.793-0.916) | 0.750 | 0.962 | 0.797 |
|  |  | External validation | 0.652 (0.536-0.767) | 0.742 | 0.870 | 0.371 |
|  | XGBoost | Training | 0.949 (0.932-0.965) | 0.702 | 0.905 | 0.845 |
|  |  | Internal validation | 0.870 (0.810-0.931) | 0.629 | 0.830 | 0.785 |
|  |  | External validation | 0.609 (0.466-0.752) | 0.774 | 0.435 | 0.814 |
|  | LightGBM | Training | 0.978 (0.963-0.992) | 0.956 | 0.938 | 0.972 |
|  |  | Internal validation | 0.884 (0.826-0.943) | 0.811 | 0.868 | 0.785 |
|  |  | External validation | 0.539 (0.393-0.685) | 0.602 | 0.349 | 0.853 |
| **Inception v3** | SVM | Training | 0.954 (0.934-0.974) | 0.882 | 0.924 | 0.902 |
|  |  | Internal validation | 0.935 (0.896-0.974) | 0.826 | 0.906 | 0.848 |
|  |  | External validation | 0.816 (0.691-0.941) | 0.806 | 0.696 | 0.886 |
|  | KNN | Training | 0.905 (0.880-0.929) | 0.823 | 0.886 | 0.731 |
|  |  | Internal validation | 0.897 (0.844-0.950) | 0.833 | 0.811 | 0.859 |
|  |  | External validation | 0.755 (0.629-0.882) | 0.785 | 0.609 | 0.843 |
|  | DecisionTrees | Training | 1.000 | 1.000 | 1.000 | 1.000 |
|  |  | Internal validation | 0.653 (0.571-0.736) | 0.674 | 0.547 | 1.000 |
|  |  | External validation | 0.444 (0.357-0.531) | 0.602 | 1.000 | 1.000 |
|  | RF | Training | 0.955 (0.939-0.972) | 0.899 | 0.981 | 0.796 |
|  |  | Internal validation | 0.742 (0.666-0.819) | 0.682 | 0.887 | 0.548 |
|  |  | External validation | 0.562 (0.429-0.696) | 0.677 | 0.348 | 1.000 |
|  | ExtraTrees | Training | 1.000 | 1.000 | 1.000 | 1.000 |
|  |  | Internal validation | 0.729 (0.647-0.811) | 0.705 | 0.792 | 0.595 |
|  |  | External validation | 0.651 (0.500-0.802) | 0.817 | 0.522 | 1.000 |
|  | XGBoost | Training | 0.921 (0.899-0.942) | 0.662 | 0.843 | 0.829 |
|  |  | Internal validation | 0.890 (0.830-0.950) | 0.682 | 0.792 | 0.861 |
|  |  | External validation | 0.786 (0.664-0.908) | 0.753 | 0.826 | 0.671 |
|  | LightGBM | Training | 0.990 (0.984-0.995) | 0.939 | 0.952 | 0.946 |
|  |  | Internal validation | 0.870 (0.807-0.934) | 0.818 | 0.755 | 0.886 |
|  |  | External validation | 0.689 (0.573-0.805) | 0.667 | 0.739 | 0.657 |
