# Supplementary figures and images for: Deep learning model for diagnosing early gastric cancer using preoperative computed tomography images
Source: Front Oncol. 2022 Nov 30;12:1065934. doi: 10.3389/fonc.2022.1065934 (PMC9748811; doi:10.3389/fonc.2022.1065934)

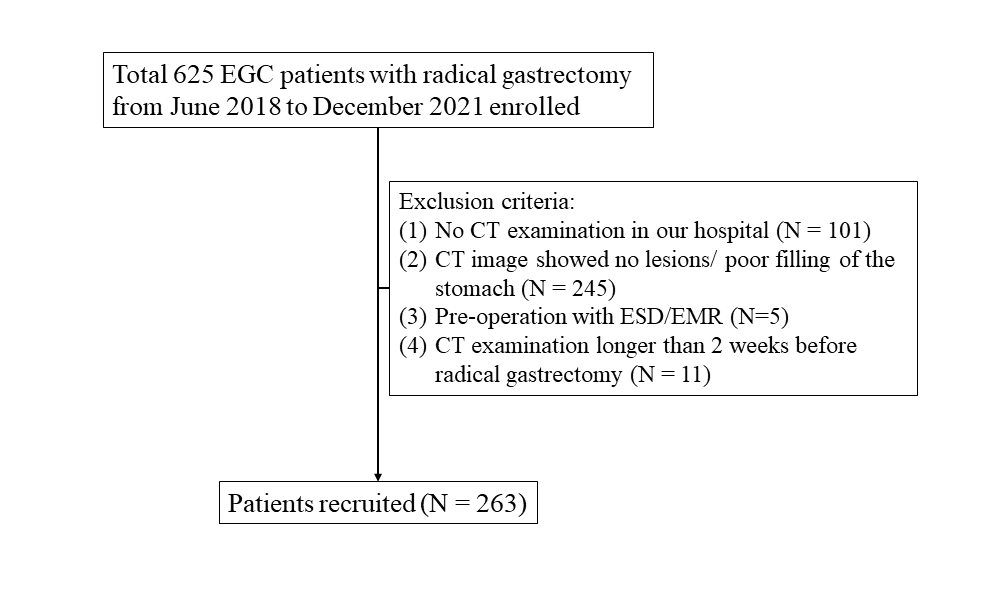

Supplement: Supplementary Figure 1 — The inclusion criteria and exclusion criteria for the patients. EGC, early gastric cancer; CT, computed tomography; ESD, endoscopic submucosal dissection. [file DataSheet_1.zip › Figure S1.TIF]

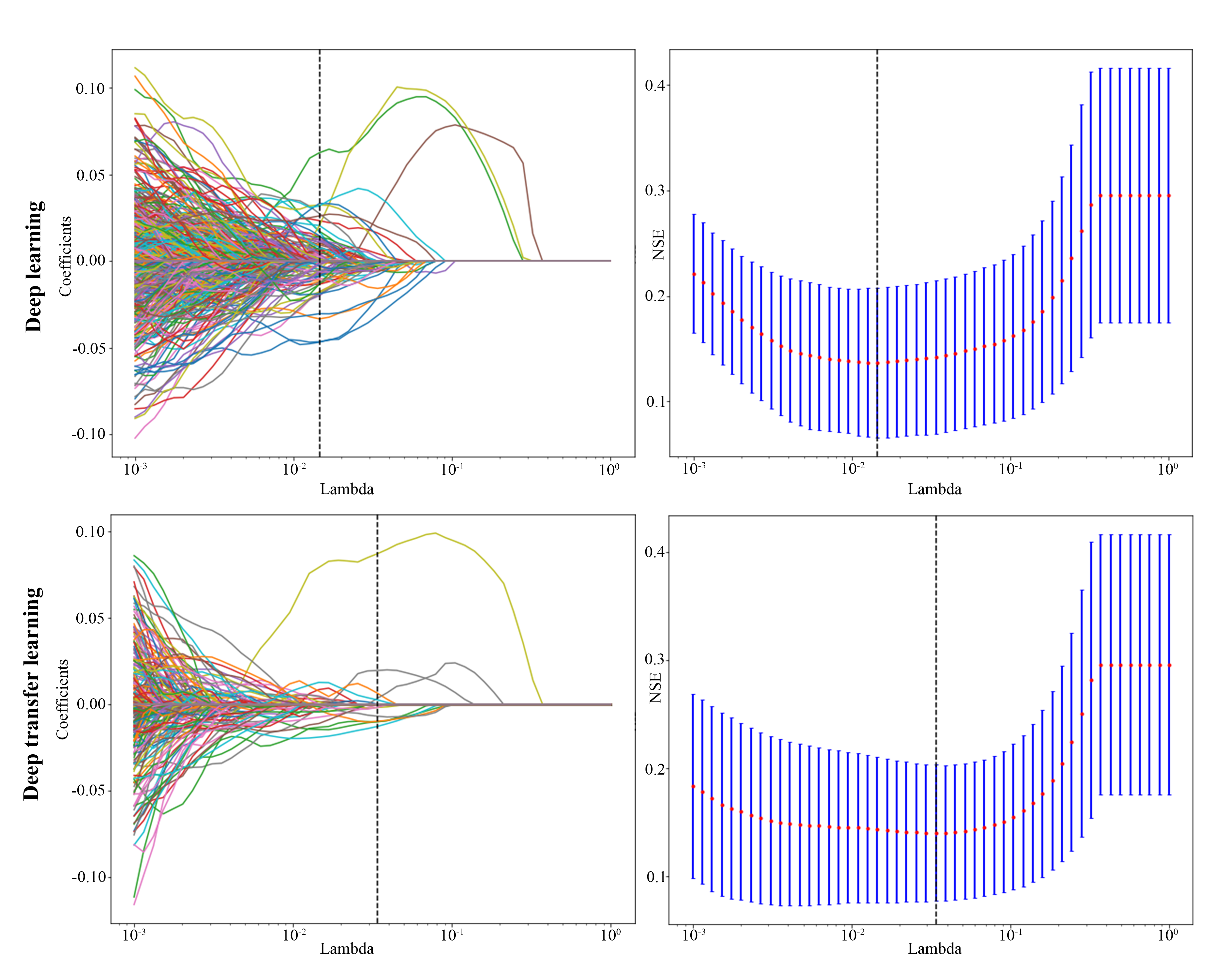

Supplement: Supplementary Figure 1 — The inclusion criteria and exclusion criteria for the patients. EGC, early gastric cancer; CT, computed tomography; ESD, endoscopic submucosal dissection. [file DataSheet_1.zip › Figure S3.TIF]

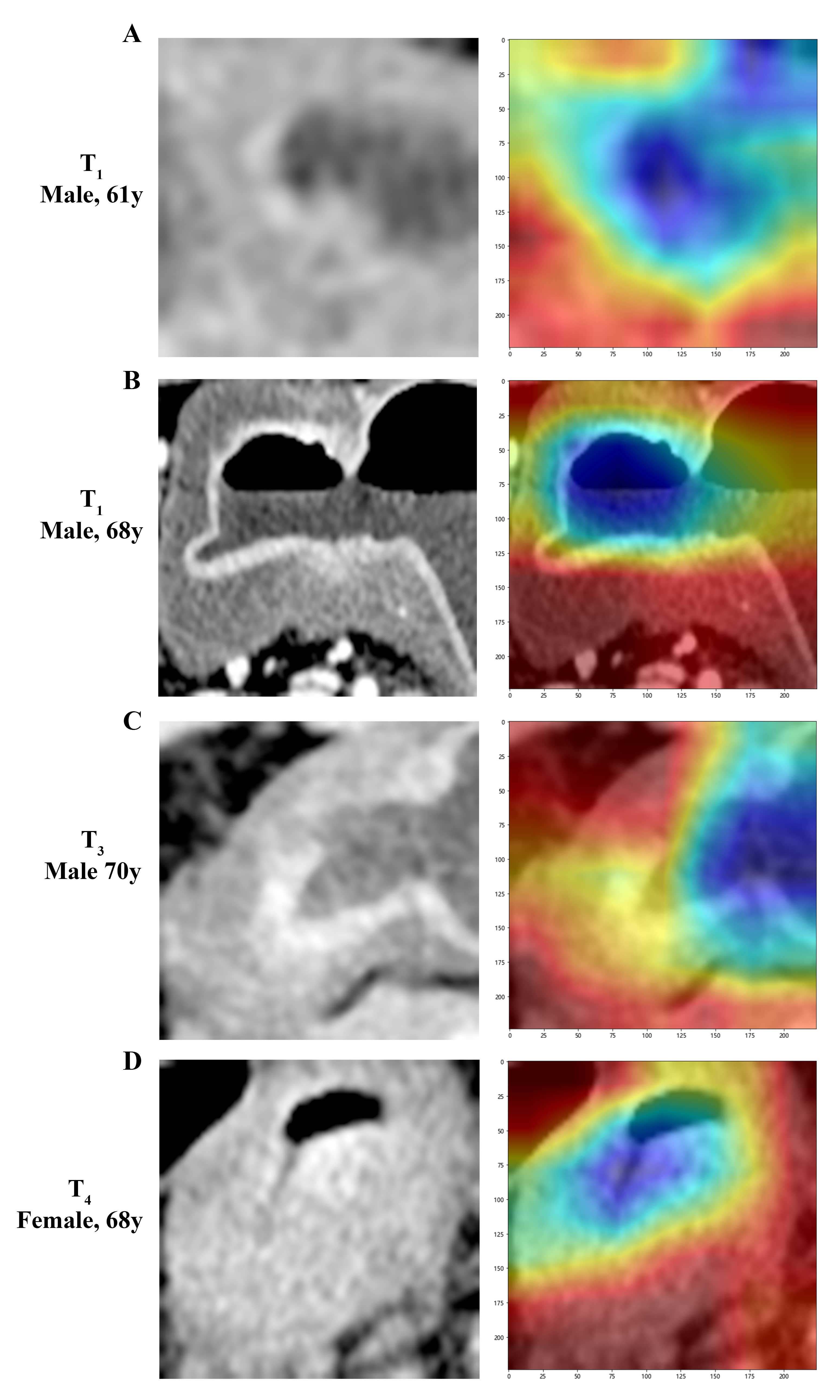

Supplement: Supplementary Figure 1 — The inclusion criteria and exclusion criteria for the patients. EGC, early gastric cancer; CT, computed tomography; ESD, endoscopic submucosal dissection. [file DataSheet_1.zip › Figure S4.TIF]
